# COMPILATION OF CONVERSION COEFFICIENTS FOR THE DOSE TO THE LENS OF THE EYE

**DOI:** 10.1093/rpd/ncw194

**Published:** 2016-08-19

**Authors:** R. Behrens

**Affiliations:** 1 Physikalisch-Technische Bundesanstalt, Bundesallee 100, D-38116Braunschweig, Germany

## Abstract

A compilation of fluence-to-absorbed dose conversion coefficients for the dose to the lens of the eye is presented. The compilation consists of both previously published data and newly calculated values: photon data (5 keV–50 MeV for both kerma approximation and full electron transport), electron data (10 keV–50 MeV), and positron data (1 keV–50 MeV) – neutron data will be published separately. Values are given for angles of incidence from 0° up to 90° in steps of 15° and for rotational irradiation. The data presented can be downloaded from this article's website and they are ready for use by Report Committee (RC) 26. This committee has been set up by the International Commission on Radiation Units and Measurements (ICRU) and is working on a ‘proposal for a redefinition of the operational quantities for external radiation exposure’.

## INTRODUCTION

The International Commission on Radiological Protection (ICRP) has published a set of *Conversion Coefficients for Radiological Protection Quantities for External Radiation Exposures*, ICRP 116^(1)^. Dose limits are set in terms of these protection quantities, while dose measurements are performed in terms of operational quantities defined by the International Commission on Radiation Units and Measurements (ICRU)^(2–6)^. In order to resolve this and in order to overcome some shortcomings of the current operational quantities, such as limited applicability at higher energies, the ICRU has initiated a report committee (RC 26) to develop a ‘proposal for a redefinition of the operational quantities for external radiation exposure’^(7)^. This committee is considering a redefinition of the operational quantities by using conversion coefficients based on the protection quantities^(8)^. Therefore, conversion coefficients for the whole body, for the lens of the eye and for local skin are necessary not only for a limited number of geometries such as those contained in ICRP 116^(1)^ (‘anterior–posterior’ AP, ‘lateral’ LAT, ‘posterior–anterior’ PA, ‘rotational’ ROT, and ‘isotropic’ ISO, see Figure 3.2 in ICRP 116) but for type tests of dosemeters also for angles of incidence from 0° up to 90° in steps of 15°. This work presents such data for the dose to the lens of the eye.

## CALCULATIONS

### Eye and body phantoms

**Figure 1.**
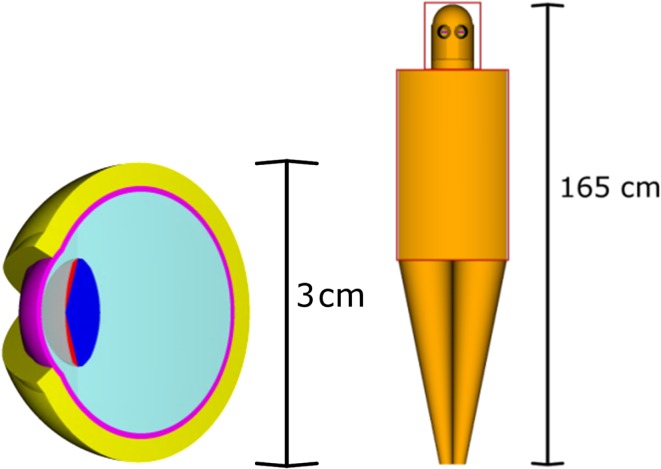
Detailed view of the eye phantom (left: the radiation sensitive part (red) and the rest of the lens (dark blue)) and the complete phantom including two eyes (right) used for the calculations. A broad parallel beam impinges on the head and the trunk (red frames in the right part).

The dimensions of the human eye relevant to radiation protection have been known for a long time^(9)^. Based on this information, a stylised eye phantom has been set up for the calculation of dose conversion coefficients^(10, 11)^, see Figure 1. This phantom contains the part of the lens that is sensitive to radiation and the part that is insensitive as separate regions. Conversion coefficients for photons and electrons^(11, 12)^ have been calculated for both the radiation sensitive part of the lens and the complete lens. Additionally, conversion coefficients for neutrons^(13)^ have been calculated for the sensitive part. ICRP 116 only provides data for the complete lens although the possibility of using the radiation sensitive part is discussed in its Annex^(1)^. In this work both the dose to the radiation sensitive part and the complete lens are presented.

### Geometries

Calculations have been performed in vacuum for radiation incidence from 0° up to 90° in steps of 15° with the rotational axis being the long axis of the phantom. A statistical uncertainty of about 1% was envisaged for relevant energies. In addition, slightly less precise calculations have been performed from 0° up to 180° in steps of 5°. Those data were averaged to obtain the values for ROT incidence. The contribution from the irradiation of the trunk is up to 90° below a few percent and up to 180° at maximum in the ten percent range order. However, the region above 90° contributes only little to the values of the ROT geometry. In conclusion, therefore, the legs have not been irradiated in the simulations as the corresponding contribution to the dose is assumed to be negligible.

### Particle types and energies

Previous publications contain data for photons^(11)^ and electrons^(12)^ but not for all angles of incidence listed above and not for all energies contained in the tables of ICRP 116. Therefore, additional calculations were performed within the frame of this work. Data were determined for the following particles and energies:

- electrons: 10 keV up to 50 MeV
- photons: 5 keV up to 50 MeV
- positrons: 1 keV up to 50 MeV.

The energy values contained in ICRP 116^(1)^ plus some additional values where the conversion coefficients strongly depend on the particle energy were used.

For photons, calculations with and without electron transport have been performed. The latter is the kerma approximation which results in charged particle equilibrium (CPE). These values are necessary for calibrations and irradiations of instruments in photon reference radiation fields in which the separate contributions of photons and (secondary) electrons is not known (this is the normal case). For such calibrations and irradiations the current practice will not change: a build-up plate made of polymethyl methacrylate (PMMA) of appropriate thickness is placed in front of the dosemeter in order to make sure CPE is present also during the irradiation^(14)^. This calculation method has been used for the conversion coefficients contained in ICRU 57^(6)^ which have been in use up to now. The drawback of this method is that no build-up plate is placed in front of the dosemeter during routine use. As a consequence, its indication is correct only if CPE (at least nearly) exists in the radiation field ‘by itself’, i.e. enough material is located between the radiation source and the dosemeter so that enough secondary electrons are produced in order to finalise the dose build-up in the detector.

Additionally, calculations including full electron transport, as is the case for the data in ICRP 116^(1)^ (i.e. no charged particle equilibrium (non-CPE)), were performed. These data are particularly to be used for the dose calculation of high energy radiation fields. In these fields, the separate contributions of photons and (secondary) electrons have to be determined in the calibration field, i.e. the spectral and angular distribution of the particle fluence. These have to be multiplied by the corresponding conversion coefficients (for photons with non-CPE and for electrons, respectively). Finally, the true dose results from the sum of these two.

### Simulations

The Monte Carlo calculations were performed using the EGSnrc code package^(15, 16)^.

As indicated in Figure 1, two broad parallel beams were simulated impinging on the head and the trunk in separate calculations, but in both calculations the complete phantom was used. Then, the two contributions were added up to the total value.

EGSnrc cannot simulate neutron particles. However, at photon energies above a few MEV, photoneutrons can be produced. In order to estimate their significance, EGSnrc has the possibility to consider photonuclear attenuation. In this case, the interaction coefficients contain the contribution from photonuclear interaction. Once this interaction occurs, the photon is discarded and no neutron is produced. As an extreme case, a photon calculation was performed at an angle of incidence of 180° (PA geometry) with and without photonuclear attenuation for several photon energies between 1 MeV and 50 MeV. Both calculations yielded the same results within the statistical uncertainty of less than 1%. In addition, a very simple geometry was used for neutron calculations with FLUKA^(17)^: a cylinder made of ICRU 4-element tissue, 15 cm in diameter and 15 cm high, with a small scoring volume (representing the lens) 3 mm below the wall's surface at the middle level of the cylinder. Calculations with mono-energetic photons and electrons with energies between 1 MeV and 50 MeV incident from different angles showed that the dose due to neutrons is several orders of magnitude smaller than the dose due to the photons and electrons themselves^(18)^. Thus, it was concluded that no significant number of photoneutrons is produced and, consequently, EGSnrc can be used for calculations up to 50 MeV particles.

## RESULTS AND DISCUSSION

### Results for the sensitive part of the lens

**Figure 2.**
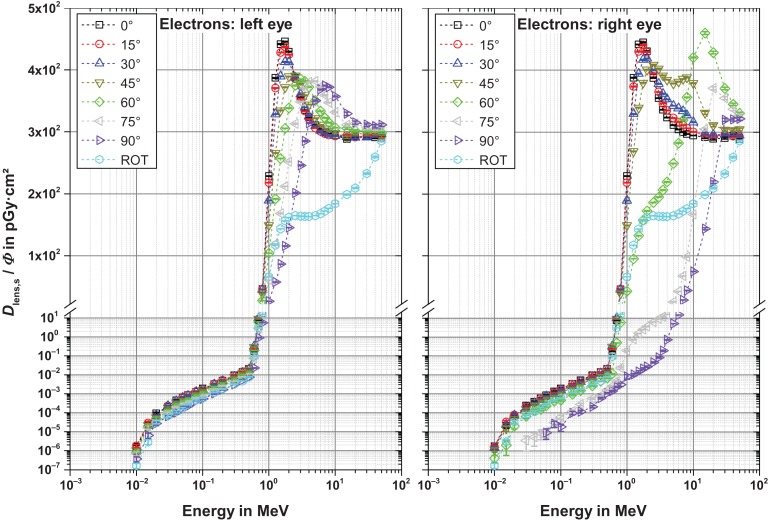
Fluence-to-absorbed dose values for the radiation sensitive cells for electrons for normal, ROT and oblique incidence from the left. Note that the ordinates are broken and the labels apply for both graphs.

**Figure 3.**
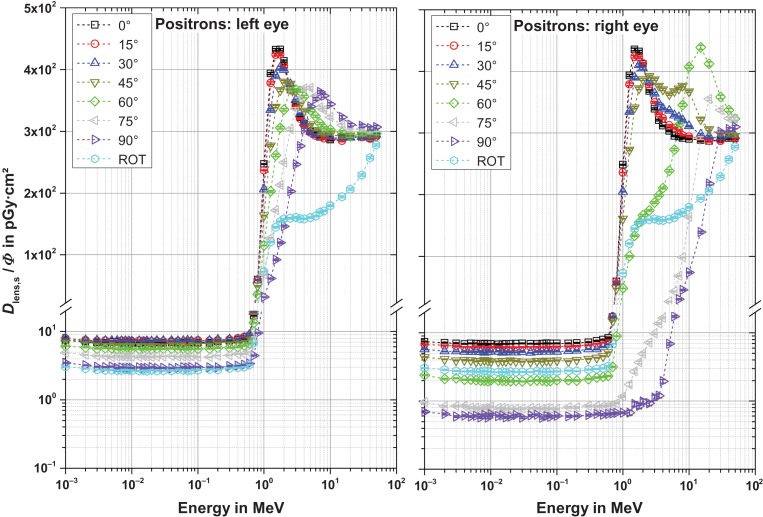
Fluence-to-absorbed dose values for the radiation sensitive cells for positrons for normal, ROT and oblique incidence from the left. Note that the ordinates are broken and the labels apply for both graphs.

**Figure 4.**
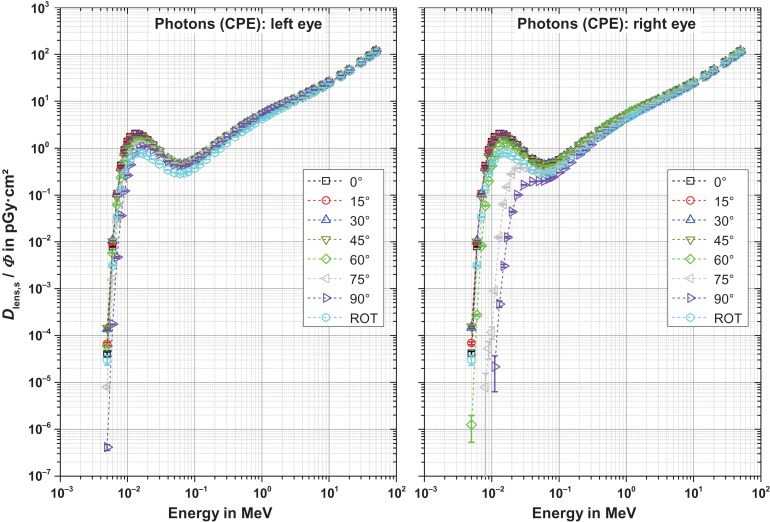
Fluence-to-absorbed dose values for the radiation sensitive cells for photons without secondary electron transport (CPE) for normal, ROT and oblique incidence from the left. Note that the labels apply for both graphs.

**Figure 5.**
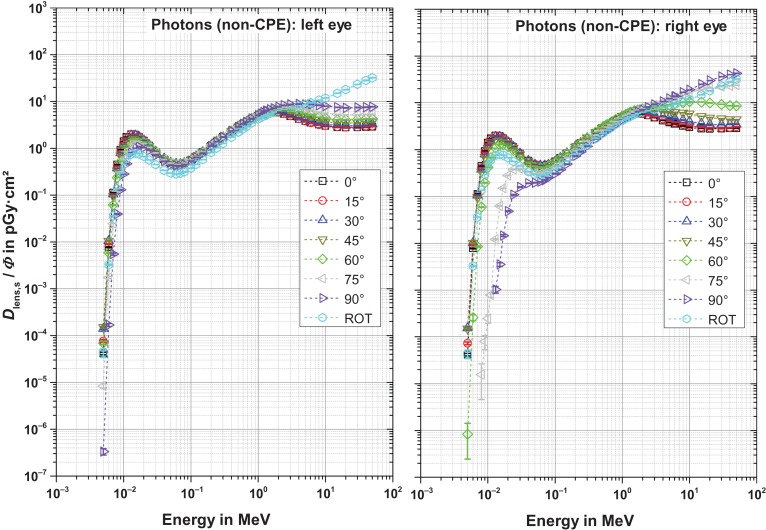
Fluence-to-absorbed dose values for the radiation sensitive cells for photons with secondary electron transport (non-CPE) for normal, ROT and oblique incidence from the left. Note that the labels apply for both graphs.

Figures 2–5 show the fluence-to-absorbed dose values for the sensitive cells for electrons, positrons and photons for CPE and non-CPE for the left and the right lens – with radiation incidence from the left (perspective of the phantom). The uncertainty bars denote the statistical one sigma uncertainties of the simulations. The non-statistical uncertainty contributions are assumed to be of the order of the interaction coefficients for the primary particles which depend on both the particle type and its energy and are not further considered here. The total uncertainty comprises the geometrical sum of the statistical and the non-statistical contribution.

### Discussion for the sensitive part of the lens

#### Electrons (Figure 2)

**Figure 6.**
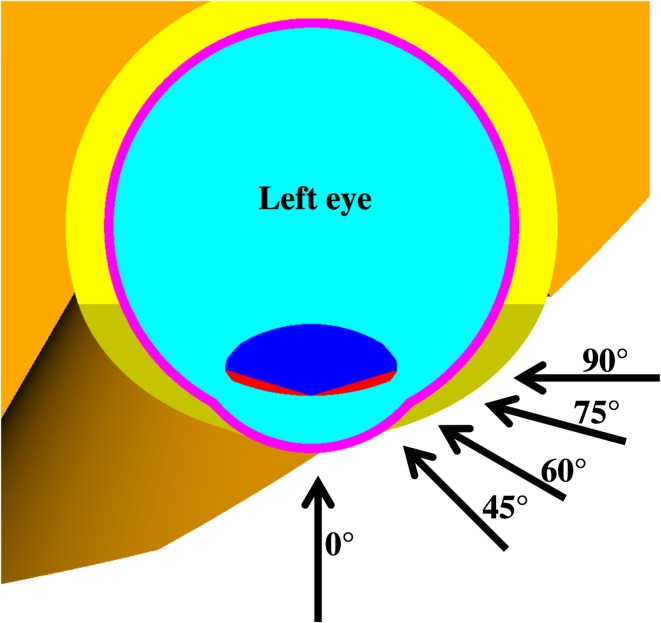
Top view of the left eye cut at its central level. The radiation sensitive part (red) and the rest of the lens (dark blue) can be seen. The arrows indicate examples of radiation incidences of broad parallel radiation beams.

Below about 0.7 MeV the electron's energy is not sufficient to propagate into the eye lens which is located about 3 mm deep in the eye. Thus, only bremsstrahlung contributes to the dose, resulting in rather small values. Above that energy, the dose per fluence rapidly increases with energy and reaches a maximum level (due to the dose build-up) at about 1.5 MeV for normal incidence (*α* = 0°). For larger angles of incidence the maximum level is shifted to higher energies, which are less pronounced and broader in terms of energy (especially for the left lens). This is because the lens is rather shallow, i.e. in the zero degree direction, the sensitive part of the lens (complete lens) has an extension of only about 0.6 (4.2) mm while in the 90° direction, it is quite extended with 10 mm, see Figure 6. Thus, at larger angles, electrons of different energy have their dose maximum within the rather extended lens (resulting in a broad maximum) while at normal incidence, only electrons with about 1.5 MeV have their dose maximum within the rather shallow lens. For ROT incidence, no dose maximum is present as here the dose is averaged over all angles of incidence (also from the back where almost only bremsstrahlung contributes).

**Figure 7.**
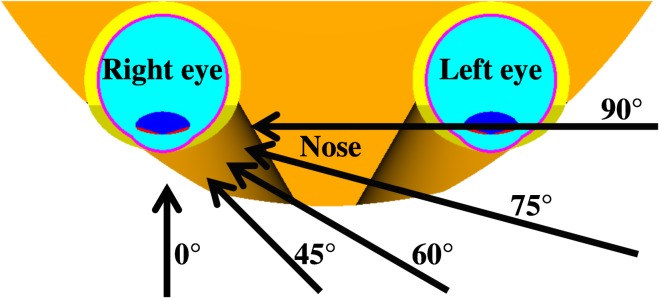
Top view of the two eyes cut at their central level. The arrows indicate examples of radiation incidences of broad parallel radiation beams.

For the right lens, up to *α* = 30°, the behaviour is rather similar (see right part of Figure 2). Above 30°, the curves are slightly different. In particular, the nose has to be penetrated, see Figure 7, partly at 60° (resulting in a very broad maximum) and totally at 75° and 90° (resulting in maxima at higher energies).

#### Positrons (Figure 3)

For positrons, the behaviour of the dose per fluence is quite similar to that of electrons, except that below about 0.7 MeV, the contribution due to annihilation photons dominates instead of bremsstrahlung photons. This contribution is independent of the positron's initial energy as the same number of annihilation photons is always produced with 511 keV, namely two per incident positron. Only the position of production depends on the angle of incidence. For example, the positrons from 90° produce the annihilation photons at quite a large distance from the right eye, see Figure 7. Therefore, the contribution of the annihilation photons is smaller than that of the positrons incident at smaller angles (see right part of Figure 3).

#### Photons, CPE (Figure 4)

For CPE and up to 90°, the dose per fluence of the left lens is nearly independent of the angle of incidence. Only for ROT incidence and between energies of 10–300 keV is it slightly smaller. The reason for this is that here the photons from the back only contribute little due to the absorption in the head. For the right lens above 60°, a stronger dependence on the angle of incidence is observed (right part of Figure 4). This is because the photons have to penetrate the nose and the left part of the head at 75° and 90°, respectively, where more absorption occurs. Above about 1 MeV nearly no difference is present compared to the left lens (as absorption does not play a significant role).

#### Photons, non-CPE (Figure 5)

Without charged particle equilibrium (non-CPE), the behaviour of the dose per fluence is quite similar to that with CPE below about 1 MeV. The reason for this is that the dose build-up is completed in the lens and CPE is achieved also in the non-CPE calculations. Above about 1 MeV, the dose build-up in the lens is not reached and the resulting dose values are smaller, depending on the amount of material in front of the lens. This amount increases with an increasing angle of incidence, especially for the right lens (see Figure 7). Therefore, the values for ROT incidence and larger angles are more similar (i.e. larger) to those of the CPE calculations than the ones for small angles of incidence.

### Results for the complete lens

**Figure 8.**
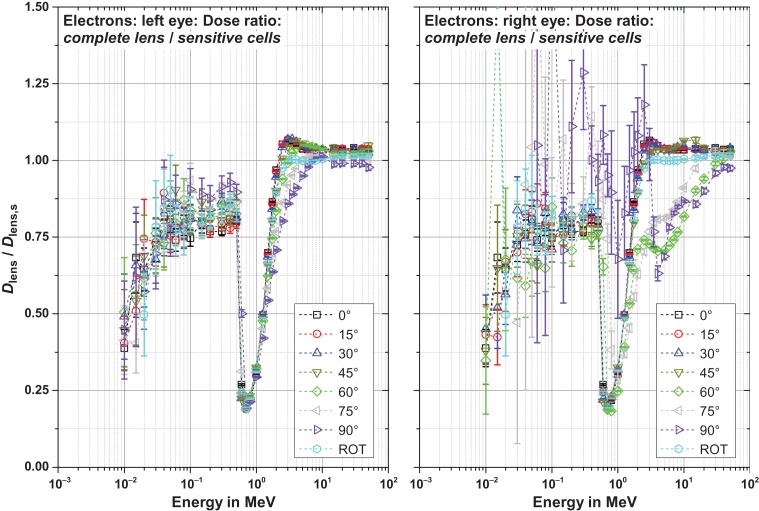
Ratio of the dose to the complete lens and the sensitive cells for electrons for radiation incidence from the left.

**Figure 9.**
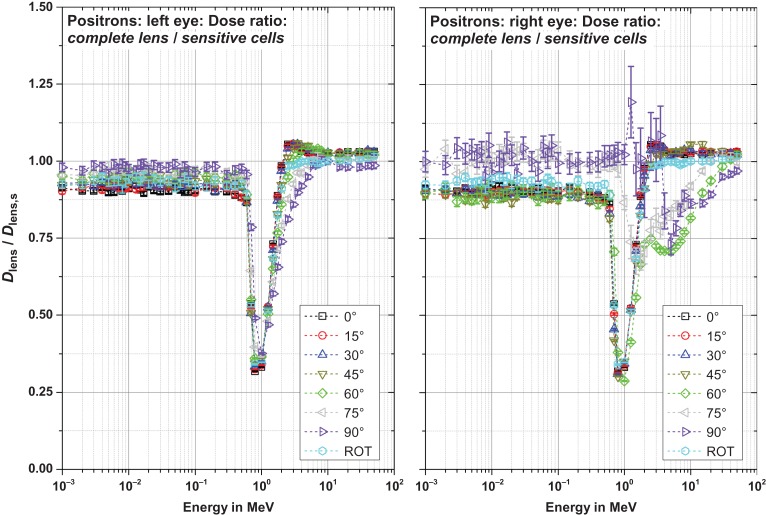
Ratio of the dose to the complete lens and the sensitive cells for positrons for radiation incidence from the left.

**Figure 10.**
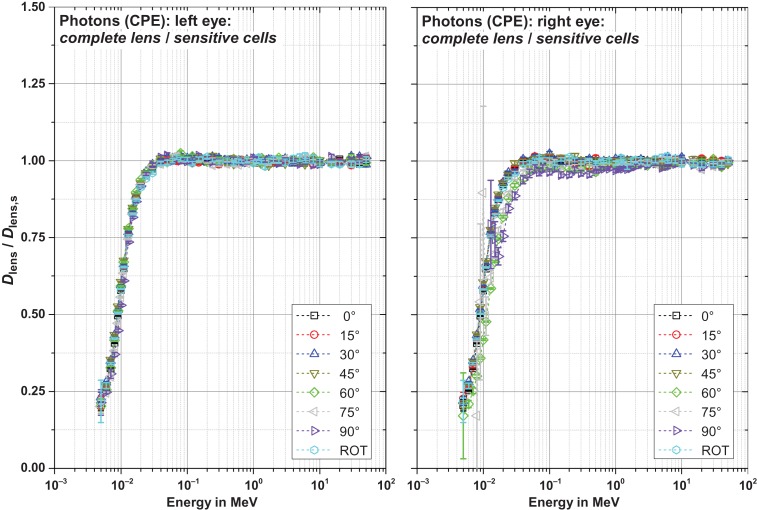
Ratio of the dose to the complete lens and the sensitive cells for photons without secondary electron transport (CPE) for radiation incidence from the left.

**Figure 11.**
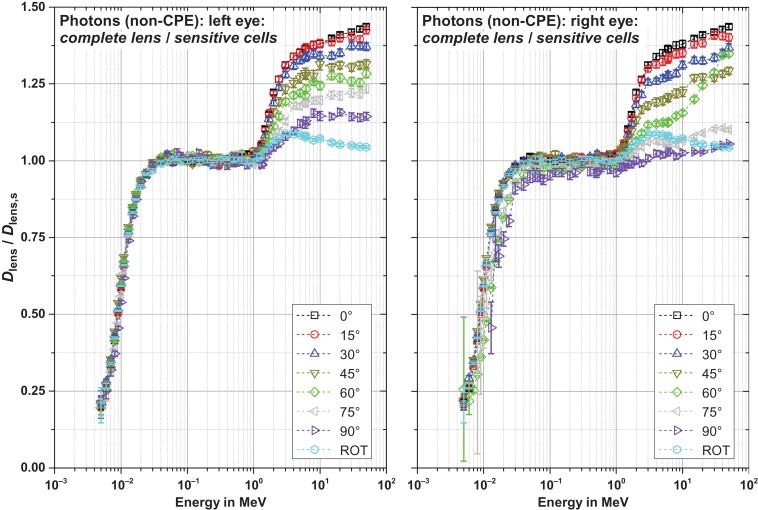
Ratio of the dose to the complete lens and the sensitive cells for photons with secondary electron transport (non-CPE) for radiation incidence from the left.

Figures 8–11 show the ratio of the dose to the complete lens and that of the sensitive cells for electrons, positrons and photons for CPE and non-CPE for the left and the right lens – with radiation incident from the left (perspective of the phantom).

### Discussion for the ratio of the dose to the complete lens and the dose to the sensitive cells

#### Electrons (Figure 8)

Since the sensitive cells are located in the front of the lens, the dose to the complete lens (i.e. averaged over the complete lens) is usually smaller than the dose to the sensitive cells (i.e. averaged over the sensitive cells only).

Below 0.7 MeV, only bremsstrahlung contributes to the dose (i.e. photons). Thus, the ratio is quite small at low energies such as 10 keV but significantly increased at 0.7 MeV.

Slightly above 0.7 MeV, the ratio strongly decreases as the electrons penetrate into the sensitive cells but not into the rear part of the lens resulting in significantly larger doses to the sensitive cells compared to the dose to the complete lens. With further increasing energy, the electrons also penetrate into the rear part of the lens and the ratio approaches unity.

At electron energies above 2 MeV the dose maximum is located in the rear part of the lens (due to the dose build-up). Therefore, the dose to the complete lens is slightly larger than the dose to the sensitive cells.

The behaviour described is quite similar for the left and the right lens. Only the statistical uncertainties are much larger for the right lens for energies below 2 MeV as radiation incidence from the left was simulated.

#### Positrons (Figure 9)

Above 0.7 MeV the shape of the ratio curves of positrons equals that of the electrons – as this is the same for the curves of the conversion coefficients (see Figures 2 and 3). Below 0.7 MeV, the behaviour is quite different. The dominating effect contributing to the dose is the annihilation radiation: 511 keV photons are produced, resulting in similar doses in the sensitive part of the lens and the complete lens. Consequently, the ratio is almost unity for all energies below 0.7 MeV.

#### Photons, CPE (Figure 10)

Above about 40 keV, the photon absorption is quite similar for both the sensitive cells and the rear part of the lens. Therefore, the dose in the sensitive cells and the complete lens is quite similar resulting in a ratio of nearly unity.

With decreasing photon energy the absorption significantly increases in the rear part of the lens (especially for small angles of incidence) resulting in a ratio smaller than unity.

#### Photons, non-CPE (Figure 11)

Below about 1 MeV, the ratios of non-CPE and CPE behave quite similar. Above that energy, for the non-CPE case the dimension of the lens is not sufficient to complete the dose build-up especially not in the sensitive cells. This results in ratios larger than unity. The ratio is the larger the smaller the angle of incidence (i.e. maximal at 0° radiation incidence) and larger for the left lens for oblique radiation incidence as much less material is located in front of the left lens compared to the right lens (due to the radiation incidence from the left).

### Dissemination of results

RC 26 considers using the maximum of the dose in the two lenses for each energy and angle of incidence in order to ensure conservatism. This is different to the approach followed in ICRP 116^(1)^ and to previously published data^(11, 12)^ where, for each energy and each angle of incidence, the mean value of the two lenses is used. In addition, using the maximum between the dose to the sensitive cells and the dose to the complete lens is under discussion. Therefore, in this work, the necessary information for both approaches is provided. The maximum values of the dose in the two lenses are given in the Appendix for the dose to the radiation sensitive cells (Tables A1–A6) and for the dose to the complete lens (Tables A7–A12). In addition to these data, separate sets of values for the left and the right lens for the sensitive cells and for the complete lens are provided in separate ASCII files accompanying this publication. Finally, a dataset containing the maximum of the left and the right lens of both the sensitive cells and the complete lens (i.e. for each particle type and energy, the maximum of these four values) is provided.

## COMPARISON WITH DATA IN ICRP 116

Some data comparable to of those contained in this work have been published in ICRP 116^(1)^. Therefore, in the following, the corresponding data from this work and from ICRP 116 are compared. In ICRP 116 data from different phantoms, i.e. the reference voxel phantoms^(19)^ and the phantom used in this work, are given as reference conversion coeﬃcients depending on the particle type, energy and irradiation geometry. The data from the stylised phantom are contained for:

- electrons for AP geometry for energies up to 10 MeV. However, these data are based on a phantom consisting of only one eye^(10)^ as the data based on the complete phantom described in this paper^(12)^ were not available at the time of issuing ICRP 116. In addition, the data from the stylised phantom were used for:
- photons for AP, PA, LAT, and ROT geometries for energies up to 2 MeV. Here, the same phantom as described in this paper was used^(11)^.

For all other energies and irradiation geometries, as well as other particle types (i.e. positrons, protons, muons, pions, helium ions), the data from the reference voxel phantoms^(19)^ (average of the data for the male and female voxel phantom) were used.

These different phantoms have to be considered when comparing the data from this work and those contained in ICRP 116.

**Figure 12.**
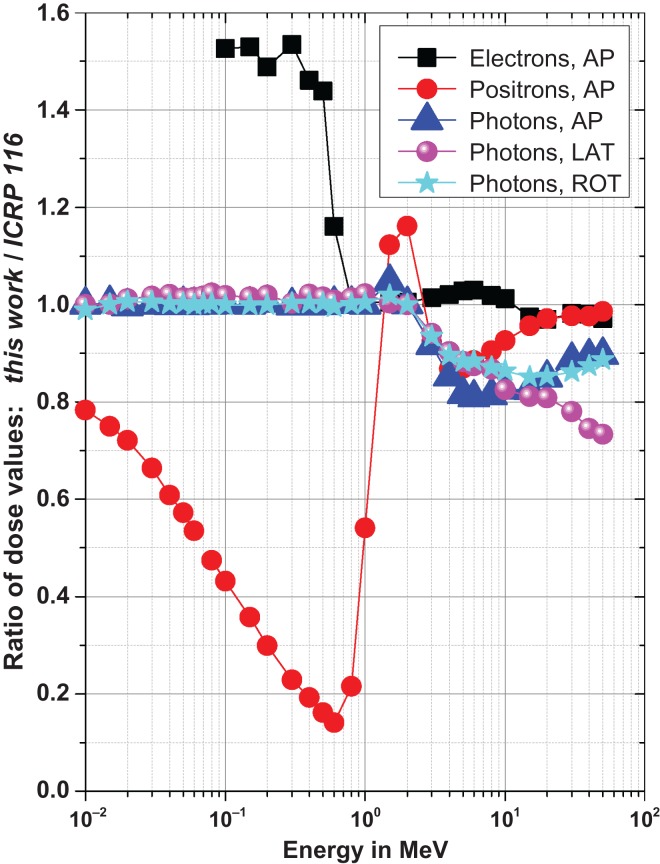
Ratio of the dose values of this work and of ICRP 116.

Figure 12 shows the ratio of the dose to the complete lens (mean values of both eyes) of this work and of ICRP 116 for those particle types, geometries and energies available in both data sets. The following features can be seen:

- Electrons, AP: below about 0.7 MeV (only bremsstrahlung contributes to the dose), the values obtained in this work are larger by about a factor of 1.5. This is because in this work the whole stylised phantom was used while the data in ICRP 116 are based on a single stylised eye. This results in less photon backscatter in the eye compared to the whole phantom. Above 0.7 MeV, the ratio is approximately unity, i.e. values from this work and ICRP 116 are nearly equal as the dose in both phantoms, only one eye vs. the whole phantom, is dominated by electrons which produce no significant backscatter in the rear part of the head.
- Positrons, AP: ICRP 116 contains data based on the voxel phantoms. However, the limited resolution of the voxels results in an inaccurate representation of the eye. As a consequence, parts of the lens are not covered by any tissue – which is, of course, not realistic. Therefore, positrons with energies below 0.7 MeV penetrate into the lens and produce a significant dose – which, in turn, is much larger than the dose in the stylised phantom used in this work where only annihilation photons contribute. Above that energy, the ratio is about unity (within about ±20%), as the positrons penetrate into the lens and produce significant doses in both phantoms.
- Photons, AP, LAT and ROT: up to 2 MeV, both works are based on the same stylised phantom. Therefore, the ratio is unity – apart from small variations as the data in ICRP 116 are smoothed. Above that energy, ICRP 116 contains data based on the voxel phantoms. These data are about 20% larger than those from this work (resulting in a ratio smaller than unity). One reason might be that the lenses in the voxel phantoms are smaller than the lenses in the stylised phantom. Therefore, complete dose build-up is reached in the voxel phantoms at smaller energies (resulting in larger doses) than in the stylised phantom.

In conclusion, it is recommended that ICRP moves from using the voxel phantoms to using the stylised phantom described in this work for the calculation of the dose to the lens of the eye for all particle types, energies and geometries. The main argument for this is that the voxel phantoms are too coarse (due to the finite size of the voxels) leading to an unrealistic eye phantom – and, in turn, leading to unrealistic dose values (as seen in the case of positrons).

## CONCLUSIONS

The fluence-to-absorbed dose values for the lens of the eye presented in this paper are ready for use by ICRU for a redefinition of the operational quantities based on protection quantities. For convenience, the data can be downloaded from this article's website. They are given for the maximum value of the two lenses and, in addition, for the left and right lens for the sensitive cells and the complete lens, separately.

## Supplementary Material

Supplementary Data

## APPENDIX A. CALCULATED VALUES OF THE ABSORBED DOSE PER INCIDENT FLUENCE

Tables A1–A4 provide fluence-to-dose conversion coefficients for the dose to the radiation sensitive cells for electrons, positrons and photons for CPE and non-CPE conditions. The values stated are the maximum value of the two lenses for each energy and angle of incidence – except for 0° and ROT, where it is the mean value of both lenses – as for these geometries, the dose in the two lenses is always the same (apart from statistical fluctuations).

For convenience, Tables A5 and A6 provide air-kerma-to-dose conversion coefficients for the dose to the cells sensitive to radiation for photons for CPE and non-CPE conditions, respectively. They result from the fluence-to-dose values (Tables A3 and A4) by division with the kerma factor:

$$\frac{D_{\text{lens},s}}{K_{a}}=\frac{D_{\text{lens},s}/\Phi }{K_{a}/\Phi }=\frac{D_{\text{lens},s}/\Phi }{\left(\mu_{\mathrm{en}}/\rho \right)\cdot E}$$

where *K*_a_ is the collision air kerma and *μ*_en_/*ρ* is the energy absorption coefficient^(20)^ for mono-energetic photons of energy *E*.

In addition, the respective data are shown for the dose to the complete lens in Tables A7–A12.

The statistical one sigma uncertainties of the values shown in the tables are of the order of 1–1.5% except for very small energies and large angles of incidence where they increase up to a maximum of about 30%.

**Table A1.** Absorbed dose per electron fluence for the radiation sensitive part of the lens, *D*_lens,s_/*Φ*, for mono-energetic particles incident at different angles *α* and the ROT geometry. Each value is the maximum value of the two lenses; except for 0° and ROT, the mean of the two lenses is given.

Electron energy (MeV)	Absorbed dose per fluence to the radiation sensitive part of the lens, *D*_lens,s_/*Φ*, (pGy cm²) for a radiation incidence at *α*							ROT geometry
	0°	15°	30°	45°	60°	75°	90°	
0.01	1.676E−06	1.813E−06	1.434E−06	1.156E−06	7.548E−07	7.039E−07	3.741E−07	1.668E−07
0.015	2.263E−05	3.337E−05	2.337E−05	2.223E−05	1.960E−05	1.659E−05	6.434E−06	2.947E−06
0.02	8.150E−05	7.792E−05	7.247E−05	5.807E−05	4.987E−05	3.241E−05	2.726E−05	3.396E−05
0.03	2.232E−04	2.304E−04	2.252E−04	1.889E−04	1.258E−04	1.124E−04	6.596E−05	7.017E−05
0.04	4.162E−04	3.398E−04	4.038E−04	3.495E−04	2.715E−04	1.998E−04	1.017E−04	1.135E−04
0.05	6.066E−04	6.749E−04	6.162E−04	5.094E−04	4.075E−04	3.058E−04	1.857E−04	1.725E−04
0.06	8.474E−04	8.680E−04	7.801E−04	6.152E−04	5.565E−04	3.874E−04	2.242E−04	2.832E−04
0.08	1.306E−03	1.333E−03	1.233E−03	1.082E−03	8.324E−04	6.107E−04	4.210E−04	4.596E−04
0.1	1.921E−03	1.805E−03	1.661E−03	1.566E−03	1.200E−03	7.965E−04	5.479E−04	5.857E−04
0.15	3.244E−03	3.358E−03	3.109E−03	2.471E−03	2.235E−03	1.651E−03	1.023E−03	1.198E−03
0.2	5.040E−03	5.210E−03	4.602E−03	4.068E−03	3.467E−03	2.588E−03	1.639E−03	1.773E−03
0.3	9.576E−03	9.075E−03	7.912E−03	7.829E−03	6.021E−03	4.518E−03	3.110E−03	3.197E−03
0.4	1.409E−02	1.469E−02	1.285E−02	1.253E−02	1.002E−02	7.883E−03	5.094E−03	4.826E−03
0.5	2.059E−02	2.056E−02	1.914E−02	1.756E−02	1.411E−02	1.134E−02	7.662E−03	7.777E−03
0.6	1.750E−01	2.322E−01	3.173E−01	2.969E−01	1.863E−01	7.494E−02	2.319E−02	9.442E−02
0.7	7.555E+00	8.463E+00	9.598E+00	8.881E+00	6.082E+00	2.891E+00	8.979E−01	3.077E+00
0.8	4.616E+01	4.661E+01	4.567E+01	3.931E+01	2.771E+01	1.498E+01	5.509E+00	1.537E+01
1	2.288E+02	2.184E+02	1.894E+02	1.496E+02	1.044E+02	6.114E+01	2.657E+01	6.640E+01
1.25	3.874E+02	3.735E+02	3.294E+02	2.690E+02	1.920E+02	1.190E+02	5.772E+01	1.174E+02
1.5	4.417E+02	4.300E+02	3.942E+02	3.394E+02	2.571E+02	1.685E+02	8.674E+01	1.437E+02
1.75	4.453E+02	4.408E+02	4.168E+02	3.795E+02	3.057E+02	2.121E+02	1.159E+02	1.565E+02
2	4.299E+02	4.291E+02	4.189E+02	4.007E+02	3.385E+02	2.519E+02	1.456E+02	1.621E+02
2.5	3.873E+02	3.942E+02	4.000E+02	4.087E+02	3.751E+02	3.131E+02	2.016E+02	1.649E+02
3	3.552E+02	3.647E+02	3.778E+02	4.024E+02	3.851E+02	3.487E+02	2.461E+02	1.641E+02
3.5	3.355E+02	3.464E+02	3.619E+02	3.915E+02	3.818E+02	3.703E+02	2.853E+02	1.637E+02
4	3.235E+02	3.351E+02	3.530E+02	3.847E+02	3.723E+02	3.802E+02	3.140E+02	1.634E+02
5	3.111E+02	3.227E+02	3.424E+02	3.784E+02	3.509E+02	3.819E+02	3.504E+02	1.650E+02
6	3.043E+02	3.162E+02	3.384E+02	3.815E+02	3.334E+02	3.729E+02	3.687E+02	1.681E+02
7	3.001E+02	3.111E+02	3.340E+02	3.859E+02	3.226E+02	3.594E+02	3.749E+02	1.722E+02
8	2.972E+02	3.064E+02	3.273E+02	3.885E+02	3.561E+02	3.472E+02	3.722E+02	1.764E+02
10	2.945E+02	3.004E+02	3.159E+02	3.790E+02	4.204E+02	3.319E+02	3.573E+02	1.843E+02
15	2.902E+02	2.953E+02	2.975E+02	3.311E+02	4.601E+02	3.114E+02	3.294E+02	1.990E+02
20	2.908E+02	2.929E+02	2.954E+02	3.110E+02	4.283E+02	3.709E+02	3.151E+02	2.094E+02
30	2.915E+02	2.958E+02	2.958E+02	3.037E+02	3.705E+02	3.548E+02	3.199E+02	2.328E+02
40	2.925E+02	2.953E+02	2.957E+02	3.031E+02	3.448E+02	3.369E+02	3.204E+02	2.596E+02
50	2.902E+02	2.932E+02	2.967E+02	3.044E+02	3.305E+02	3.269E+02	3.215E+02	2.856E+02

**Table A2.** Absorbed dose per positron fluence for the radiation sensitive part of the lens, *D*_lens,s_/*Φ*, for mono-energetic particles incident at different angles *α* and the ROT geometry. Each value is the maximum value of the two lenses; except for 0° and ROT, the mean of the two lenses is given.

Positron energy (MeV)	Absorbed dose per fluence to the radiation sensitive part of the lens, *D*_lens,s_/*Φ*, (pGy cm²) for a radiation incidence at *α*							ROT geometry
	0°	15°	30°	45°	60°	75°	90°	
0.001	7.433E+00	7.939E+00	7.893E+00	7.345E+00	6.134E+00	4.906E+00	3.470E+00	3.060E+00
0.002	7.165E+00	7.467E+00	7.434E+00	6.978E+00	5.876E+00	4.582E+00	3.206E+00	2.871E+00
0.003	6.993E+00	7.261E+00	7.387E+00	6.713E+00	5.614E+00	4.426E+00	3.052E+00	2.762E+00
0.004	6.881E+00	7.343E+00	7.282E+00	6.643E+00	5.699E+00	4.363E+00	3.007E+00	2.725E+00
0.005	7.025E+00	7.172E+00	7.287E+00	6.720E+00	5.674E+00	4.241E+00	3.067E+00	2.742E+00
0.006	6.971E+00	7.179E+00	7.329E+00	6.587E+00	5.538E+00	4.330E+00	2.914E+00	2.697E+00
0.007	6.851E+00	7.313E+00	7.222E+00	6.653E+00	5.584E+00	4.328E+00	2.981E+00	2.692E+00
0.008	6.924E+00	7.306E+00	7.210E+00	6.655E+00	5.509E+00	4.266E+00	2.950E+00	2.770E+00
0.009	6.843E+00	7.299E+00	7.137E+00	6.596E+00	5.567E+00	4.299E+00	2.944E+00	2.727E+00
0.01	6.869E+00	7.258E+00	7.181E+00	6.610E+00	5.519E+00	4.311E+00	2.938E+00	2.674E+00
0.013	6.777E+00	7.318E+00	7.204E+00	6.619E+00	5.527E+00	4.232E+00	2.978E+00	2.651E+00
0.015	6.878E+00	7.290E+00	7.077E+00	6.546E+00	5.648E+00	4.376E+00	2.922E+00	2.690E+00
0.017	6.957E+00	7.194E+00	7.231E+00	6.558E+00	5.545E+00	4.302E+00	2.938E+00	2.634E+00
0.02	6.795E+00	7.131E+00	7.173E+00	6.588E+00	5.494E+00	4.244E+00	3.008E+00	2.723E+00
0.024	6.926E+00	7.219E+00	7.282E+00	6.572E+00	5.595E+00	4.298E+00	2.917E+00	2.667E+00
0.03	6.945E+00	7.180E+00	7.271E+00	6.488E+00	5.594E+00	4.312E+00	2.943E+00	2.685E+00
0.04	6.945E+00	7.155E+00	7.256E+00	6.686E+00	5.615E+00	4.391E+00	2.906E+00	2.672E+00
0.05	6.874E+00	7.200E+00	7.197E+00	6.763E+00	5.550E+00	4.287E+00	2.919E+00	2.772E+00
0.06	6.908E+00	7.044E+00	7.278E+00	6.736E+00	5.606E+00	4.307E+00	2.996E+00	2.710E+00
0.07	6.993E+00	7.172E+00	7.355E+00	6.476E+00	5.523E+00	4.333E+00	2.960E+00	2.680E+00
0.08	6.964E+00	7.203E+00	7.211E+00	6.798E+00	5.610E+00	4.376E+00	3.008E+00	2.707E+00
0.1	6.970E+00	7.430E+00	7.257E+00	6.708E+00	5.572E+00	4.254E+00	3.004E+00	2.725E+00
0.15	6.943E+00	7.299E+00	7.302E+00	6.675E+00	5.626E+00	4.235E+00	2.983E+00	2.791E+00
0.2	7.134E+00	7.415E+00	7.595E+00	6.931E+00	5.762E+00	4.432E+00	2.993E+00	2.749E+00
0.3	7.292E+00	7.611E+00	7.663E+00	6.971E+00	5.801E+00	4.498E+00	3.120E+00	2.900E+00
0.4	7.601E+00	7.966E+00	7.710E+00	7.116E+00	5.883E+00	4.701E+00	3.162E+00	2.959E+00
0.5	7.913E+00	8.345E+00	8.111E+00	7.355E+00	6.227E+00	4.698E+00	3.242E+00	3.023E+00
0.6	8.443E+00	8.695E+00	8.855E+00	8.110E+00	6.667E+00	4.983E+00	3.392E+00	3.262E+00
0.7	1.711E+01	1.845E+01	1.954E+01	1.804E+01	1.344E+01	8.332E+00	4.465E+00	6.911E+00
0.8	5.955E+01	5.972E+01	5.861E+01	5.031E+01	3.652E+01	2.146E+01	9.545E+00	2.017E+01
1	2.480E+02	2.371E+02	2.063E+02	1.640E+02	1.153E+02	6.880E+01	3.182E+01	7.283E+01
1.25	3.935E+02	3.791E+02	3.350E+02	2.771E+02	2.031E+02	1.274E+02	6.182E+01	1.210E+02
1.5	4.339E+02	4.242E+02	3.900E+02	3.414E+02	2.612E+02	1.721E+02	9.221E+01	1.450E+02
1.75	4.323E+02	4.245E+02	4.104E+02	3.725E+02	3.057E+02	2.131E+02	1.198E+02	1.539E+02
2	4.133E+02	4.130E+02	4.052E+02	3.872E+02	3.351E+02	2.498E+02	1.465E+02	1.581E+02
2.5	3.680E+02	3.759E+02	3.830E+02	3.923E+02	3.681E+02	3.035E+02	2.029E+02	1.593E+02
3	3.412E+02	3.497E+02	3.642E+02	3.870E+02	3.676E+02	3.370E+02	2.466E+02	1.609E+02
3.5	3.220E+02	3.313E+02	3.482E+02	3.766E+02	3.656E+02	3.533E+02	2.762E+02	1.587E+02
4	3.124E+02	3.240E+02	3.397E+02	3.709E+02	3.564E+02	3.694E+02	3.031E+02	1.585E+02
5	3.017E+02	3.126E+02	3.362E+02	3.628E+02	3.340E+02	3.703E+02	3.404E+02	1.604E+02
6	2.969E+02	3.083E+02	3.288E+02	3.679E+02	3.245E+02	3.573E+02	3.524E+02	1.630E+02
7	2.925E+02	3.043E+02	3.257E+02	3.722E+02	3.128E+02	3.389E+02	3.638E+02	1.692E+02
8	2.921E+02	2.964E+02	3.206E+02	3.765E+02	3.456E+02	3.349E+02	3.578E+02	1.711E+02
10	2.887E+02	2.942E+02	3.113E+02	3.677E+02	4.077E+02	3.231E+02	3.443E+02	1.796E+02
15	2.903E+02	2.907E+02	2.975E+02	3.222E+02	4.389E+02	3.099E+02	3.230E+02	1.941E+02
20	2.894E+02	2.911E+02	2.941E+02	3.062E+02	4.123E+02	3.553E+02	3.106E+02	2.065E+02
30	2.902E+02	2.908E+02	2.919E+02	2.976E+02	3.588E+02	3.373E+02	3.092E+02	2.294E+02
40	2.906E+02	2.944E+02	2.950E+02	2.976E+02	3.372E+02	3.239E+02	3.061E+02	2.574E+02
50	2.926E+02	2.914E+02	2.954E+02	2.952E+02	3.231E+02	3.233E+02	3.100E+02	2.780E+02

**Table A3.** Absorbed dose per photon fluence (CPE) for the radiation sensitive part of the lens, *D*_lens,s_/*Φ*, for mono-energetic particles incident at different angles *α* and the ROT geometry. Each value is the maximum value of the two lenses; except for 0° and ROT, the mean of the two lenses is given.

Photon energy (MeV)	Absorbed dose per fluence to the radiation sensitive part of the lens, *D*_lens,s_/*Φ*, (pGy cm²) for a radiation incidence at *α*							ROT geometry
	0°	15°	30°	45°	60°	75°	90°	
0.005	4.097E−05	6.907E−05	1.483E−04	1.564E−04	5.948E−05	7.938E−06	4.113E−07	3.062E−05
0.006	7.937E−03	8.866E−03	1.061E−02	1.018E−02	5.725E−03	1.558E−03	1.743E−04	3.131E−03
0.007	1.067E−01	1.064E−01	1.046E−01	9.158E−02	6.220E−02	2.668E−02	4.672E−03	3.388E−02
0.008	4.252E−01	4.123E−01	3.788E−01	3.244E−01	2.349E−01	1.235E−01	3.646E−02	1.292E−01
0.009	9.033E−01	8.756E−01	7.974E−01	6.774E−01	5.207E−01	3.199E−01	1.225E−01	2.852E−01
0.01	1.376E+00	1.336E+00	1.228E+00	1.063E+00	8.583E−01	5.786E−01	2.629E−01	4.525E−01
0.011	1.729E+00	1.687E+00	1.567E+00	1.386E+00	1.151E+00	8.450E−01	4.425E−01	5.921E−01
0.013	2.024E+00	1.997E+00	1.899E+00	1.739E+00	1.529E+00	1.253E+00	7.870E−01	7.593E−01
0.015	1.978E+00	1.955E+00	1.885E+00	1.774E+00	1.632E+00	1.409E+00	1.017E+00	7.971E−01
0.017	1.805E+00	1.788E+00	1.740E+00	1.674E+00	1.550E+00	1.400E+00	1.106E+00	7.723E−01
0.02	1.494E+00	1.487E+00	1.472E+00	1.426E+00	1.352E+00	1.264E+00	1.071E+00	6.873E−01
0.024	1.162E+00	1.172E+00	1.154E+00	1.138E+00	1.106E+00	1.050E+00	9.275E−01	5.793E−01
0.03	8.534E−01	8.485E−01	8.504E−01	8.498E−01	8.240E−01	7.934E−01	7.281E−01	4.586E−01
0.04	5.888E−01	5.953E−01	5.973E−01	5.923E−01	5.874E−01	5.768E−01	5.303E−01	3.426E−01
0.05	4.892E−01	4.880E−01	4.943E−01	5.005E−01	4.970E−01	4.800E−01	4.462E−01	2.993E−01
0.06	4.508E−01	4.524E−01	4.612E−01	4.636E−01	4.587E−01	4.414E−01	4.275E−01	2.856E−01
0.07	4.554E−01	4.628E−01	4.611E−01	4.614E−01	4.587E−01	4.461E−01	4.311E−01	2.921E−01
0.08	4.809E−01	4.862E−01	4.840E−01	4.868E−01	4.766E−01	4.767E−01	4.602E−01	3.141E−01
0.1	5.576E−01	5.593E−01	5.682E−01	5.695E−01	5.619E−01	5.585E−01	5.419E−01	3.750E−01
0.12	6.617E−01	6.696E−01	6.683E−01	6.726E−01	6.624E−01	6.655E−01	6.425E−01	4.559E−01
0.15	8.355E−01	8.395E−01	8.484E−01	8.407E−01	8.443E−01	8.366E−01	8.072E−01	5.780E−01
0.2	1.138E+00	1.144E+00	1.157E+00	1.167E+00	1.151E+00	1.147E+00	1.120E+00	8.079E−01
0.24	1.378E+00	1.399E+00	1.398E+00	1.385E+00	1.419E+00	1.403E+00	1.380E+00	9.963E−01
0.3	1.751E+00	1.757E+00	1.762E+00	1.783E+00	1.797E+00	1.762E+00	1.733E+00	1.297E+00
0.4	2.295E+00	2.374E+00	2.330E+00	2.372E+00	2.391E+00	2.343E+00	2.286E+00	1.749E+00
0.5	2.818E+00	2.823E+00	2.876E+00	2.929E+00	3.009E+00	2.864E+00	2.843E+00	2.228E+00
0.511	2.906E+00	2.915E+00	2.948E+00	2.997E+00	2.977E+00	2.898E+00	2.923E+00	2.258E+00
0.6	3.348E+00	3.368E+00	3.420E+00	3.478E+00	3.489E+00	3.395E+00	3.378E+00	2.644E+00
0.662	3.644E+00	3.649E+00	3.694E+00	3.780E+00	3.785E+00	3.661E+00	3.650E+00	2.917E+00
0.8	4.242E+00	4.326E+00	4.306E+00	4.384E+00	4.408E+00	4.319E+00	4.309E+00	3.462E+00
1	5.093E+00	5.096E+00	5.120E+00	5.240E+00	5.305E+00	5.120E+00	5.091E+00	4.236E+00
1.117	5.580E+00	5.572E+00	5.709E+00	5.683E+00	5.742E+00	5.572E+00	5.538E+00	4.632E+00
1.2	5.814E+00	5.931E+00	5.930E+00	5.976E+00	6.028E+00	5.896E+00	5.855E+00	4.871E+00
1.3	6.229E+00	6.271E+00	6.293E+00	6.415E+00	6.435E+00	6.246E+00	6.259E+00	5.250E+00
1.33	6.321E+00	6.361E+00	6.352E+00	6.448E+00	6.515E+00	6.373E+00	6.351E+00	5.386E+00
1.5	6.894E+00	6.976E+00	6.914E+00	7.016E+00	7.095E+00	6.902E+00	6.890E+00	5.975E+00
1.7	7.467E+00	7.579E+00	7.588E+00	7.679E+00	7.775E+00	7.551E+00	7.530E+00	6.487E+00
2	8.386E+00	8.445E+00	8.466E+00	8.531E+00	8.623E+00	8.418E+00	8.346E+00	7.227E+00
2.4	9.406E+00	9.533E+00	9.523E+00	9.705E+00	9.720E+00	9.479E+00	9.603E+00	8.319E+00
3	1.098E+01	1.103E+01	1.105E+01	1.109E+01	1.125E+01	1.091E+01	1.089E+01	9.821E+00
4	1.321E+01	1.331E+01	1.342E+01	1.332E+01	1.339E+01	1.319E+01	1.318E+01	1.180E+01
5	1.543E+01	1.538E+01	1.548E+01	1.570E+01	1.561E+01	1.529E+01	1.550E+01	1.376E+01
6	1.740E+01	1.742E+01	1.753E+01	1.764E+01	1.752E+01	1.744E+01	1.748E+01	1.565E+01
6.129	1.762E+01	1.773E+01	1.767E+01	1.798E+01	1.806E+01	1.743E+01	1.760E+01	1.608E+01
8	2.148E+01	2.167E+01	2.143E+01	2.153E+01	2.160E+01	2.149E+01	2.160E+01	1.934E+01
10	2.540E+01	2.545E+01	2.574E+01	2.550E+01	2.549E+01	2.559E+01	2.564E+01	2.360E+01
15	3.594E+01	3.613E+01	3.566E+01	3.574E+01	3.586E+01	3.550E+01	3.523E+01	3.316E+01
20	4.610E+01	4.634E+01	4.686E+01	4.657E+01	4.667E+01	4.653E+01	4.635E+01	4.346E+01
30	6.958E+01	6.974E+01	6.961E+01	6.927E+01	6.870E+01	7.003E+01	6.855E+01	6.454E+01
40	9.398E+01	9.335E+01	9.349E+01	9.397E+01	9.486E+01	9.328E+01	9.210E+01	8.724E+01
50	1.186E+02	1.193E+02	1.202E+02	1.188E+02	1.186E+02	1.166E+02	1.170E+02	1.090E+02

**Table A4.** Absorbed dose per photon fluence (non-CPE) for the radiation sensitive part of the lens, *D*_lens,s_/*Φ*, for mono-energetic particles incident at different angles *α* and the ROT geometry. Each value is the maximum value of the two lenses; except for 0° and ROT, the mean of the two lenses is given.

Photon energy (MeV)	Absorbed dose per fluence to the radiation sensitive part of the lens, *D*_lens,s_/*Φ*, (pGy cm²) for a radiation incidence at *α*							ROT geometry
	0°	15°	30°	45°	60°	75°	90°	
0.005	4.166E−05	7.651E−05	1.536E−04	1.516E−04	6.904E−05	8.410E−06	3.344E−07	4.344E−05
0.006	7.745E−03	9.299E−03	1.059E−02	1.052E−02	5.870E−03	1.735E−03	1.697E−04	3.277E−03
0.007	1.127E−01	1.070E−01	1.051E−01	9.547E−02	6.157E−02	2.483E−02	5.498E−03	3.642E−02
0.008	4.460E−01	4.118E−01	3.768E−01	3.370E−01	2.366E−01	1.217E−01	3.949E−02	1.371E−01
0.009	9.371E−01	8.787E−01	8.000E−01	7.040E−01	5.195E−01	3.183E−01	1.296E−01	2.938E−01
0.01	1.416E+00	1.335E+00	1.230E+00	1.093E+00	8.491E−01	5.735E−01	2.824E−01	4.628E−01
0.011	1.749E+00	1.682E+00	1.567E+00	1.413E+00	1.148E+00	8.448E−01	4.679E−01	6.025E−01
0.013	2.009E+00	1.995E+00	1.894E+00	1.725E+00	1.535E+00	1.234E+00	8.089E−01	7.577E−01
0.015	1.940E+00	1.955E+00	1.889E+00	1.750E+00	1.622E+00	1.410E+00	1.014E+00	7.909E−01
0.017	1.758E+00	1.786E+00	1.744E+00	1.635E+00	1.555E+00	1.400E+00	1.090E+00	7.621E−01
0.02	1.453E+00	1.486E+00	1.478E+00	1.387E+00	1.363E+00	1.265E+00	1.035E+00	6.741E−01
0.024	1.143E+00	1.164E+00	1.165E+00	1.116E+00	1.103E+00	1.041E+00	9.075E−01	5.606E−01
0.03	8.258E−01	8.539E−01	8.445E−01	8.168E−01	8.266E−01	8.021E−01	7.081E−01	4.481E−01
0.04	5.801E−01	5.962E−01	6.034E−01	5.865E−01	5.863E−01	5.737E−01	5.303E−01	3.391E−01
0.05	4.768E−01	4.918E−01	4.944E−01	4.919E−01	4.939E−01	4.778E−01	4.388E−01	2.955E−01
0.06	4.446E−01	4.625E−01	4.693E−01	4.628E−01	4.515E−01	4.498E−01	4.274E−01	2.827E−01
0.07	4.522E−01	4.600E−01	4.634E−01	4.595E−01	4.557E−01	4.476E−01	4.226E−01	2.913E−01
0.08	4.783E−01	4.778E−01	4.889E−01	4.836E−01	4.871E−01	4.802E−01	4.519E−01	3.152E−01
0.1	5.574E−01	5.622E−01	5.626E−01	5.716E−01	5.657E−01	5.489E−01	5.398E−01	3.768E−01
0.12	6.655E−01	6.702E−01	6.687E−01	6.789E−01	6.604E−01	6.610E−01	6.401E−01	4.522E−01
0.15	8.353E−01	8.415E−01	8.410E−01	8.451E−01	8.480E−01	8.380E−01	7.908E−01	5.767E−01
0.2	1.133E+00	1.145E+00	1.169E+00	1.161E+00	1.147E+00	1.136E+00	1.121E+00	8.102E−01
0.24	1.386E+00	1.398E+00	1.402E+00	1.398E+00	1.398E+00	1.384E+00	1.392E+00	1.014E+00
0.3	1.744E+00	1.751E+00	1.773E+00	1.833E+00	1.788E+00	1.741E+00	1.746E+00	1.275E+00
0.4	2.281E+00	2.305E+00	2.310E+00	2.370E+00	2.365E+00	2.315E+00	2.362E+00	1.742E+00
0.5	2.801E+00	2.827E+00	2.874E+00	2.894E+00	2.960E+00	2.833E+00	2.848E+00	2.205E+00
0.511	2.873E+00	2.901E+00	2.912E+00	3.020E+00	3.019E+00	2.833E+00	2.855E+00	2.249E+00
0.6	3.326E+00	3.323E+00	3.396E+00	3.442E+00	3.465E+00	3.354E+00	3.333E+00	2.623E+00
0.662	3.580E+00	3.646E+00	3.629E+00	3.750E+00	3.730E+00	3.594E+00	3.658E+00	2.896E+00
0.8	4.176E+00	4.221E+00	4.287E+00	4.311E+00	4.443E+00	4.270E+00	4.328E+00	3.484E+00
1	4.970E+00	4.965E+00	5.079E+00	5.267E+00	5.243E+00	5.143E+00	5.078E+00	4.193E+00
1.117	5.397E+00	5.406E+00	5.501E+00	5.487E+00	5.555E+00	5.584E+00	5.463E+00	4.556E+00
1.2	5.656E+00	5.658E+00	5.709E+00	5.760E+00	5.923E+00	5.815E+00	5.895E+00	4.843E+00
1.3	5.788E+00	5.852E+00	5.906E+00	6.084E+00	6.310E+00	6.064E+00	6.265E+00	5.100E+00
1.33	5.851E+00	5.924E+00	6.051E+00	6.043E+00	6.368E+00	6.182E+00	6.379E+00	5.154E+00
1.5	5.974E+00	6.044E+00	6.183E+00	6.475E+00	6.790E+00	6.557E+00	6.758E+00	5.572E+00
1.7	6.016E+00	6.087E+00	6.326E+00	6.621E+00	7.075E+00	6.980E+00	7.136E+00	5.927E+00
2	5.763E+00	5.948E+00	6.158E+00	6.760E+00	7.441E+00	7.548E+00	7.718E+00	6.351E+00
2.4	5.410E+00	5.479E+00	5.961E+00	6.720E+00	7.771E+00	8.271E+00	8.273E+00	6.836E+00
3	4.841E+00	4.997E+00	5.511E+00	6.669E+00	8.055E+00	9.053E+00	9.199E+00	7.225E+00
4	4.200E+00	4.453E+00	5.105E+00	6.443E+00	8.602E+00	1.029E+01	1.072E+01	7.937E+00
5	3.796E+00	4.046E+00	4.796E+00	6.423E+00	9.021E+00	1.151E+01	1.238E+01	8.625E+00
6	3.533E+00	3.795E+00	4.588E+00	6.304E+00	9.529E+00	1.282E+01	1.370E+01	9.279E+00
6.129	3.518E+00	3.787E+00	4.515E+00	6.316E+00	9.556E+00	1.267E+01	1.383E+01	9.353E+00
8	3.215E+00	3.465E+00	4.294E+00	6.123E+00	1.009E+01	1.479E+01	1.681E+01	1.069E+01
10	3.020E+00	3.239E+00	3.961E+00	5.853E+00	1.057E+01	1.647E+01	1.899E+01	1.184E+01
15	2.840E+00	3.019E+00	3.601E+00	5.177E+00	1.041E+01	1.929E+01	2.501E+01	1.494E+01
20	2.796E+00	2.954E+00	3.473E+00	4.826E+00	9.986E+00	2.097E+01	3.001E+01	1.798E+01
30	2.822E+00	2.924E+00	3.434E+00	4.596E+00	9.260E+00	2.229E+01	3.644E+01	2.330E+01
40	2.863E+00	2.976E+00	3.463E+00	4.435E+00	8.712E+00	2.303E+01	4.021E+01	2.815E+01
50	2.896E+00	3.082E+00	3.442E+00	4.412E+00	8.632E+00	2.394E+01	4.294E+01	3.216E+01

**Table A5.** Absorbed dose per photon air kerma (CPE) for the radiation sensitive part of the lens, *D*_lens,s_/*K*_a_, for mono-energetic particles incident at different angles *α* and the ROT geometry. Each value is the maximum value of the two lenses; except for 0° and ROT, the mean of the two lenses is given.

Photon energy (MeV)	Absorbed dose per air kerma to the radiation sensitive part of the lens, *D*_lens,s_/*K*_a_, (Gy/Gy) for a radiation incidence at *α*							ROT geometry
	0°	15°	30°	45°	60°	75°	90°	
0.005	1.301E−06	2.194E−06	4.710E−06	4.967E−06	1.889E−06	2.521E−07	1.306E−08	9.723E−07
0.006	3.638E−04	4.063E−04	4.863E−04	4.665E−04	2.624E−04	7.141E−05	7.990E−06	1.435E−04
0.007	6.703E−03	6.682E−03	6.569E−03	5.754E−03	3.908E−03	1.676E−03	2.936E−04	2.129E−03
0.008	3.511E−02	3.404E−02	3.128E−02	2.679E−02	1.939E−02	1.020E−02	3.010E−03	1.067E−02
0.009	9.540E−02	9.248E−02	8.422E−02	7.155E−02	5.500E−02	3.378E−02	1.294E−02	3.012E−02
0.01	1.811E−01	1.759E−01	1.616E−01	1.400E−01	1.130E−01	7.616E−02	3.461E−02	5.956E−02
0.011	2.787E−01	2.720E−01	2.527E−01	2.234E−01	1.856E−01	1.362E−01	7.135E−02	9.546E−02
0.013	4.655E−01	4.595E−01	4.368E−01	4.001E−01	3.517E−01	2.883E−01	1.810E−01	1.747E−01
0.015	6.170E−01	6.098E−01	5.880E−01	5.533E−01	5.089E−01	4.394E−01	3.171E−01	2.486E−01
0.017	7.367E−01	7.299E−01	7.105E−01	6.834E−01	6.327E−01	5.717E−01	4.514E−01	3.153E−01
0.02	8.652E−01	8.610E−01	8.522E−01	8.254E−01	7.829E−01	7.317E−01	6.204E−01	3.980E−01
0.024	9.858E−01	9.943E−01	9.786E−01	9.656E−01	9.381E−01	8.904E−01	7.868E−01	4.914E−01
0.03	1.155E+00	1.149E+00	1.151E+00	1.150E+00	1.115E+00	1.074E+00	9.857E−01	6.209E−01
0.04	1.345E+00	1.359E+00	1.364E+00	1.353E+00	1.341E+00	1.317E+00	1.211E+00	7.824E−01
0.05	1.490E+00	1.486E+00	1.506E+00	1.524E+00	1.514E+00	1.462E+00	1.359E+00	9.117E−01
0.06	1.542E+00	1.548E+00	1.578E+00	1.586E+00	1.569E+00	1.510E+00	1.463E+00	9.771E−01
0.07	1.569E+00	1.595E+00	1.589E+00	1.590E+00	1.581E+00	1.537E+00	1.486E+00	1.007E+00
0.08	1.559E+00	1.576E+00	1.569E+00	1.578E+00	1.545E+00	1.545E+00	1.492E+00	1.018E+00
0.1	1.497E+00	1.502E+00	1.525E+00	1.529E+00	1.508E+00	1.499E+00	1.455E+00	1.007E+00
0.12	1.434E+00	1.451E+00	1.448E+00	1.457E+00	1.435E+00	1.442E+00	1.392E+00	9.879E−01
0.15	1.393E+00	1.400E+00	1.414E+00	1.402E+00	1.408E+00	1.395E+00	1.346E+00	9.636E−01
0.2	1.329E+00	1.336E+00	1.351E+00	1.363E+00	1.345E+00	1.340E+00	1.308E+00	9.435E−01
0.24	1.299E+00	1.318E+00	1.318E+00	1.305E+00	1.337E+00	1.322E+00	1.301E+00	9.388E−01
0.3	1.269E+00	1.273E+00	1.277E+00	1.292E+00	1.302E+00	1.277E+00	1.256E+00	9.402E−01
0.4	1.214E+00	1.256E+00	1.233E+00	1.255E+00	1.265E+00	1.240E+00	1.210E+00	9.252E−01
0.5	1.186E+00	1.188E+00	1.210E+00	1.233E+00	1.267E+00	1.205E+00	1.196E+00	9.375E−01
0.511	1.197E+00	1.201E+00	1.215E+00	1.235E+00	1.227E+00	1.194E+00	1.204E+00	9.304E−01
0.6	1.179E+00	1.186E+00	1.205E+00	1.225E+00	1.229E+00	1.196E+00	1.190E+00	9.315E−01
0.662	1.173E+00	1.175E+00	1.189E+00	1.217E+00	1.219E+00	1.179E+00	1.175E+00	9.392E−01
0.8	1.148E+00	1.171E+00	1.166E+00	1.187E+00	1.193E+00	1.169E+00	1.167E+00	9.371E−01
1	1.140E+00	1.141E+00	1.146E+00	1.173E+00	1.187E+00	1.146E+00	1.139E+00	9.480E−01
1.117	1.146E+00	1.144E+00	1.173E+00	1.167E+00	1.179E+00	1.144E+00	1.137E+00	9.512E−01
1.2	1.130E+00	1.152E+00	1.152E+00	1.161E+00	1.171E+00	1.146E+00	1.138E+00	9.464E−01
1.3	1.137E+00	1.145E+00	1.149E+00	1.171E+00	1.175E+00	1.140E+00	1.143E+00	9.586E−01
1.33	1.134E+00	1.141E+00	1.140E+00	1.157E+00	1.169E+00	1.143E+00	1.139E+00	9.662E−01
1.5	1.126E+00	1.140E+00	1.130E+00	1.146E+00	1.159E+00	1.128E+00	1.126E+00	9.764E−01
1.7	1.116E+00	1.133E+00	1.134E+00	1.148E+00	1.162E+00	1.128E+00	1.125E+00	9.694E−01
2	1.116E+00	1.124E+00	1.127E+00	1.135E+00	1.148E+00	1.120E+00	1.111E+00	9.617E−01
2.4	1.106E+00	1.121E+00	1.120E+00	1.142E+00	1.143E+00	1.115E+00	1.130E+00	9.786E−01
3	1.111E+00	1.116E+00	1.118E+00	1.122E+00	1.138E+00	1.104E+00	1.101E+00	9.934E−01
4	1.103E+00	1.111E+00	1.120E+00	1.112E+00	1.117E+00	1.101E+00	1.100E+00	9.848E−01
5	1.107E+00	1.103E+00	1.111E+00	1.126E+00	1.120E+00	1.097E+00	1.112E+00	9.873E−01
6	1.099E+00	1.100E+00	1.107E+00	1.114E+00	1.107E+00	1.102E+00	1.104E+00	9.884E−01
6.129	1.096E+00	1.102E+00	1.099E+00	1.118E+00	1.123E+00	1.084E+00	1.094E+00	1.000E+00
8	1.099E+00	1.109E+00	1.096E+00	1.101E+00	1.105E+00	1.099E+00	1.105E+00	9.893E−01
10	1.094E+00	1.096E+00	1.108E+00	1.098E+00	1.097E+00	1.102E+00	1.104E+00	1.016E+00
15	1.106E+00	1.111E+00	1.097E+00	1.099E+00	1.103E+00	1.092E+00	1.084E+00	1.020E+00
20	1.097E+00	1.103E+00	1.115E+00	1.108E+00	1.111E+00	1.108E+00	1.103E+00	1.035E+00
30	1.134E+00	1.136E+00	1.134E+00	1.128E+00	1.119E+00	1.141E+00	1.117E+00	1.052E+00
40	1.162E+00	1.154E+00	1.156E+00	1.162E+00	1.173E+00	1.153E+00	1.139E+00	1.079E+00
50	1.182E+00	1.190E+00	1.198E+00	1.184E+00	1.183E+00	1.162E+00	1.166E+00	1.087E+00

**Table A6.** Absorbed dose per photon air kerma (non-CPE) for the radiation sensitive part of the lens, *D*_lens,s_/*K*_a_, for mono-energetic particles incident at different angles *α* and the ROT geometry. Each value is the maximum value of the two lenses; except for 0° and ROT, the mean of the two lenses is given.

Photon energy (MeV)	Absorbed dose per air kerma to the radiation sensitive part of the lens, *D*_lens,s_/*K*_a_, (Gy/Gy) for a radiation incidence at *α*							ROT geometry
	0°	15°	30°	45°	60°	75°	90°	
0.005	1.323E−06	2.430E−06	4.877E−06	4.816E−06	2.193E−06	2.671E−07	1.062E−08	1.379E−06
0.006	3.550E−04	4.262E−04	4.851E−04	4.821E−04	2.690E−04	7.950E−05	7.779E−06	1.502E−04
0.007	7.082E−03	6.722E−03	6.605E−03	5.998E−03	3.868E−03	1.560E−03	3.454E−04	2.288E−03
0.008	3.683E−02	3.400E−02	3.111E−02	2.783E−02	1.953E−02	1.005E−02	3.261E−03	1.132E−02
0.009	9.897E−02	9.280E−02	8.449E−02	7.435E−02	5.487E−02	3.362E−02	1.369E−02	3.103E−02
0.01	1.864E−01	1.758E−01	1.619E−01	1.439E−01	1.118E−01	7.549E−02	3.717E−02	6.092E−02
0.011	2.820E−01	2.712E−01	2.527E−01	2.278E−01	1.851E−01	1.362E−01	7.543E−02	9.714E−02
0.013	4.621E−01	4.589E−01	4.357E−01	3.969E−01	3.531E−01	2.839E−01	1.861E−01	1.743E−01
0.015	6.050E−01	6.099E−01	5.893E−01	5.457E−01	5.059E−01	4.397E−01	3.163E−01	2.467E−01
0.017	7.176E−01	7.289E−01	7.121E−01	6.677E−01	6.348E−01	5.714E−01	4.448E−01	3.111E−01
0.02	8.415E−01	8.602E−01	8.558E−01	8.030E−01	7.891E−01	7.322E−01	5.990E−01	3.903E−01
0.024	9.700E−01	9.874E−01	9.880E−01	9.464E−01	9.355E−01	8.833E−01	7.698E−01	4.756E−01
0.03	1.118E+00	1.156E+00	1.143E+00	1.106E+00	1.119E+00	1.086E+00	9.586E−01	6.066E−01
0.04	1.325E+00	1.361E+00	1.378E+00	1.339E+00	1.339E+00	1.310E+00	1.211E+00	7.744E−01
0.05	1.452E+00	1.498E+00	1.506E+00	1.498E+00	1.505E+00	1.455E+00	1.337E+00	9.001E−01
0.06	1.521E+00	1.582E+00	1.605E+00	1.583E+00	1.545E+00	1.539E+00	1.462E+00	9.672E−01
0.07	1.558E+00	1.585E+00	1.597E+00	1.584E+00	1.571E+00	1.542E+00	1.456E+00	1.004E+00
0.08	1.550E+00	1.549E+00	1.585E+00	1.568E+00	1.579E+00	1.557E+00	1.465E+00	1.022E+00
0.1	1.496E+00	1.509E+00	1.510E+00	1.534E+00	1.519E+00	1.473E+00	1.449E+00	1.011E+00
0.12	1.442E+00	1.452E+00	1.449E+00	1.471E+00	1.431E+00	1.432E+00	1.387E+00	9.800E−01
0.15	1.393E+00	1.403E+00	1.402E+00	1.409E+00	1.414E+00	1.397E+00	1.319E+00	9.615E−01
0.2	1.324E+00	1.337E+00	1.365E+00	1.356E+00	1.340E+00	1.327E+00	1.310E+00	9.463E−01
0.24	1.306E+00	1.317E+00	1.321E+00	1.318E+00	1.317E+00	1.304E+00	1.312E+00	9.556E−01
0.3	1.264E+00	1.269E+00	1.285E+00	1.329E+00	1.296E+00	1.261E+00	1.265E+00	9.236E−01
0.4	1.207E+00	1.220E+00	1.222E+00	1.254E+00	1.251E+00	1.225E+00	1.250E+00	9.214E−01
0.5	1.179E+00	1.190E+00	1.209E+00	1.218E+00	1.246E+00	1.192E+00	1.199E+00	9.278E−01
0.511	1.184E+00	1.195E+00	1.200E+00	1.244E+00	1.244E+00	1.167E+00	1.176E+00	9.266E−01
0.6	1.172E+00	1.171E+00	1.196E+00	1.212E+00	1.221E+00	1.181E+00	1.174E+00	9.241E−01
0.662	1.153E+00	1.174E+00	1.168E+00	1.207E+00	1.201E+00	1.157E+00	1.178E+00	9.323E−01
0.8	1.130E+00	1.143E+00	1.161E+00	1.167E+00	1.203E+00	1.156E+00	1.171E+00	9.431E−01
1	1.112E+00	1.111E+00	1.137E+00	1.179E+00	1.173E+00	1.151E+00	1.136E+00	9.385E−01
1.117	1.108E+00	1.110E+00	1.130E+00	1.127E+00	1.141E+00	1.147E+00	1.122E+00	9.357E−01
1.2	1.099E+00	1.099E+00	1.109E+00	1.119E+00	1.151E+00	1.130E+00	1.145E+00	9.410E−01
1.3	1.057E+00	1.069E+00	1.078E+00	1.111E+00	1.152E+00	1.107E+00	1.144E+00	9.312E−01
1.33	1.050E+00	1.063E+00	1.085E+00	1.084E+00	1.142E+00	1.109E+00	1.144E+00	9.245E−01
1.5	9.761E−01	9.876E−01	1.010E+00	1.058E+00	1.109E+00	1.071E+00	1.104E+00	9.105E−01
1.7	8.991E−01	9.096E−01	9.453E−01	9.894E−01	1.057E+00	1.043E+00	1.066E+00	8.858E−01
2	7.670E−01	7.916E−01	8.195E−01	8.996E−01	9.903E−01	1.004E+00	1.027E+00	8.453E−01
2.4	6.364E−01	6.446E−01	7.012E−01	7.905E−01	9.141E−01	9.729E−01	9.732E−01	8.041E−01
3	4.897E−01	5.054E−01	5.574E−01	6.745E−01	8.147E−01	9.157E−01	9.304E−01	7.308E−01
4	3.505E−01	3.717E−01	4.261E−01	5.378E−01	7.180E−01	8.588E−01	8.946E−01	6.625E−01
5	2.723E−01	2.903E−01	3.440E−01	4.608E−01	6.472E−01	8.257E−01	8.878E−01	6.187E−01
6	2.232E−01	2.397E−01	2.898E−01	3.983E−01	6.020E−01	8.101E−01	8.652E−01	5.862E−01
6.129	2.188E−01	2.355E−01	2.808E−01	3.928E−01	5.943E−01	7.879E−01	8.598E−01	5.817E−01
8	1.645E−01	1.772E−01	2.197E−01	3.132E−01	5.162E−01	7.566E−01	8.599E−01	5.468E−01
10	1.300E−01	1.395E−01	1.705E−01	2.519E−01	4.549E−01	7.091E−01	8.176E−01	5.098E−01
15	8.736E−02	9.285E−02	1.108E−01	1.592E−01	3.201E−01	5.933E−01	7.694E−01	4.597E−01
20	6.656E−02	7.032E−02	8.268E−02	1.149E−01	2.377E−01	4.992E−01	7.143E−01	4.280E−01
30	4.597E−02	4.763E−02	5.594E−02	7.488E−02	1.509E−01	3.632E−01	5.936E−01	3.796E−01
40	3.540E−02	3.680E−02	4.282E−02	5.484E−02	1.077E−01	2.848E−01	4.972E−01	3.481E−01
50	2.887E−02	3.073E−02	3.432E−02	4.399E−02	8.606E−02	2.387E−01	4.281E−01	3.207E−01

**Table A7.** Absorbed dose per electron fluence for the complete lens, *D*_lens_/*Φ*, for mono-energetic particles incident at different angles *α* and the ROT geometry. Each value is the maximum value of the two lenses; except for 0° and ROT, the mean of the two lenses is given.

Electron energy (MeV)	Absorbed dose per fluence to the radiation sensitive part of the lens, *D*_lens,s_/*Φ*, (pGy cm²) for a radiation incidence at *α*							ROT geometry
	0°	15°	30°	45°	60°	75°	90°	
0.01	6.504E−07	7.335E−07	6.459E−07	4.681E−07	3.815E−07	2.912E−07	1.672E−07	3.324E−07
0.015	1.546E−05	1.472E−05	1.367E−05	1.204E−05	1.101E−05	6.714E−06	3.995E−06	4.598E−06
0.02	5.256E−05	5.212E−05	4.292E−05	4.001E−05	3.069E−05	2.006E−05	1.561E−05	1.689E−05
0.03	1.618E−04	1.690E−04	1.523E−04	1.367E−04	9.591E−05	7.958E−05	4.587E−05	5.486E−05
0.04	3.161E−04	3.036E−04	3.066E−04	2.529E−04	1.985E−04	1.554E−04	8.873E−05	9.570E−05
0.05	4.900E−04	5.093E−04	4.759E−04	3.941E−04	3.452E−04	2.360E−04	1.443E−04	1.560E−04
0.06	6.272E−04	6.420E−04	6.125E−04	5.166E−04	4.431E−04	3.054E−04	1.927E−04	2.189E−04
0.08	1.029E−03	1.049E−03	9.823E−04	8.336E−04	7.251E−04	5.148E−04	3.275E−04	3.505E−04
0.1	1.434E−03	1.468E−03	1.344E−03	1.261E−03	1.006E−03	7.439E−04	4.956E−04	4.835E−04
0.15	2.600E−03	2.663E−03	2.484E−03	2.137E−03	1.786E−03	1.368E−03	9.097E−04	9.398E−04
0.2	3.870E−03	4.046E−03	3.763E−03	3.392E−03	2.807E−03	2.176E−03	1.434E−03	1.444E−03
0.3	7.364E−03	7.184E−03	6.859E−03	6.293E−03	5.149E−03	4.068E−03	2.843E−03	2.627E−03
0.4	1.140E−02	1.145E−02	1.081E−02	1.016E−02	8.492E−03	6.676E−03	4.727E−03	4.188E−03
0.5	1.655E−02	1.634E−02	1.558E−02	1.440E−02	1.222E−02	9.687E−03	6.870E−03	6.377E−03
0.6	4.712E−02	5.679E−02	6.979E−02	6.616E−02	4.506E−02	2.340E−02	1.161E−02	2.294E−02
0.7	1.463E+00	1.647E+00	1.881E+00	1.726E+00	1.165E+00	5.487E−01	1.729E−01	5.983E−01
0.8	1.004E+01	1.031E+01	1.043E+01	9.077E+00	6.283E+00	3.276E+00	1.175E+00	3.453E+00
1	6.946E+01	6.737E+01	6.021E+01	4.859E+01	3.362E+01	1.886E+01	7.801E+00	2.077E+01
1.25	1.921E+02	1.853E+02	1.636E+02	1.317E+02	9.149E+01	5.378E+01	2.429E+01	5.714E+01
1.5	3.084E+02	2.990E+02	2.693E+02	2.233E+02	1.605E+02	9.819E+01	4.720E+01	9.533E+01
1.75	3.847E+02	3.781E+02	3.497E+02	3.024E+02	2.279E+02	1.461E+02	7.386E+01	1.260E+02
2	4.160E+02	4.123E+02	3.947E+02	3.583E+02	2.831E+02	1.935E+02	1.030E+02	1.453E+02
2.5	4.075E+02	4.120E+02	4.128E+02	4.043E+02	3.523E+02	2.699E+02	1.618E+02	1.609E+02
3	3.781E+02	3.870E+02	3.987E+02	4.111E+02	3.817E+02	3.206E+02	2.114E+02	1.640E+02
3.5	3.544E+02	3.657E+02	3.817E+02	4.050E+02	3.910E+02	3.524E+02	2.536E+02	1.641E+02
4	3.388E+02	3.508E+02	3.690E+02	3.985E+02	3.873E+02	3.700E+02	2.859E+02	1.638E+02
5	3.227E+02	3.350E+02	3.544E+02	3.919E+02	3.679E+02	3.811E+02	3.328E+02	1.648E+02
6	3.149E+02	3.279E+02	3.491E+02	3.943E+02	3.494E+02	3.764E+02	3.607E+02	1.680E+02
7	3.104E+02	3.222E+02	3.461E+02	4.005E+02	3.369E+02	3.640E+02	3.746E+02	1.721E+02
8	3.075E+02	3.179E+02	3.411E+02	4.063E+02	3.281E+02	3.527E+02	3.757E+02	1.765E+02
10	3.045E+02	3.117E+02	3.299E+02	4.033E+02	3.437E+02	3.356E+02	3.614E+02	1.852E+02
15	3.011E+02	3.059E+02	3.086E+02	3.529E+02	4.146E+02	3.146E+02	3.255E+02	2.006E+02
20	3.015E+02	3.033E+02	3.054E+02	3.233E+02	4.027E+02	3.759E+02	3.123E+02	2.124E+02
30	3.033E+02	3.044E+02	3.061E+02	3.117E+02	3.666E+02	3.666E+02	3.087E+02	2.362E+02
40	3.028E+02	3.065E+02	3.034E+02	3.127E+02	3.471E+02	3.456E+02	3.133E+02	2.644E+02
50	3.001E+02	3.014E+02	3.061E+02	3.103E+02	3.406E+02	3.319E+02	3.133E+02	2.903E+02

**Table A8.** Absorbed dose per positron fluence for the complete lens, *D*_lens_/*Φ*, for mono-energetic particles incident at different angles *α* and the ROT geometry. Each value is the maximum value of the two lenses; except for 0° and ROT, the mean of the two lenses is given.

Positron energy (MeV)	Absorbed dose per fluence to the radiation sensitive part of the lens, *D*_lens,s_/*Φ*, (pGy cm²) for a radiation incidence at *α*							ROT geometry
	0°	15°	30°	45°	60°	75°	90°	
0.001	6.789E+00	7.164E+00	7.325E+00	6.780E+00	5.839E+00	4.695E+00	3.399E+00	2.828E+00
0.002	6.473E+00	6.820E+00	6.916E+00	6.442E+00	5.508E+00	4.372E+00	3.106E+00	2.652E+00
0.003	6.320E+00	6.680E+00	6.789E+00	6.301E+00	5.383E+00	4.263E+00	3.008E+00	2.588E+00
0.004	6.289E+00	6.655E+00	6.783E+00	6.267E+00	5.368E+00	4.207E+00	2.948E+00	2.585E+00
0.005	6.305E+00	6.615E+00	6.752E+00	6.270E+00	5.328E+00	4.182E+00	2.940E+00	2.553E+00
0.006	6.262E+00	6.615E+00	6.710E+00	6.255E+00	5.244E+00	4.168E+00	2.882E+00	2.517E+00
0.007	6.271E+00	6.674E+00	6.700E+00	6.235E+00	5.280E+00	4.154E+00	2.905E+00	2.519E+00
0.008	6.255E+00	6.663E+00	6.646E+00	6.233E+00	5.309E+00	4.138E+00	2.925E+00	2.519E+00
0.009	6.251E+00	6.647E+00	6.705E+00	6.226E+00	5.304E+00	4.157E+00	2.892E+00	2.536E+00
0.01	6.255E+00	6.634E+00	6.706E+00	6.177E+00	5.329E+00	4.177E+00	2.863E+00	2.519E+00
0.013	6.251E+00	6.645E+00	6.675E+00	6.232E+00	5.259E+00	4.120E+00	2.888E+00	2.514E+00
0.015	6.238E+00	6.657E+00	6.654E+00	6.194E+00	5.308E+00	4.135E+00	2.889E+00	2.516E+00
0.017	6.230E+00	6.586E+00	6.706E+00	6.199E+00	5.299E+00	4.122E+00	2.912E+00	2.504E+00
0.02	6.228E+00	6.576E+00	6.686E+00	6.184E+00	5.279E+00	4.151E+00	2.917E+00	2.520E+00
0.024	6.265E+00	6.636E+00	6.701E+00	6.210E+00	5.299E+00	4.158E+00	2.880E+00	2.512E+00
0.03	6.256E+00	6.594E+00	6.663E+00	6.187E+00	5.264E+00	4.104E+00	2.881E+00	2.506E+00
0.04	6.265E+00	6.552E+00	6.725E+00	6.192E+00	5.284E+00	4.141E+00	2.847E+00	2.501E+00
0.05	6.290E+00	6.616E+00	6.661E+00	6.224E+00	5.310E+00	4.119E+00	2.889E+00	2.533E+00
0.06	6.259E+00	6.583E+00	6.677E+00	6.281E+00	5.282E+00	4.142E+00	2.882E+00	2.513E+00
0.07	6.285E+00	6.657E+00	6.748E+00	6.178E+00	5.313E+00	4.196E+00	2.909E+00	2.531E+00
0.08	6.258E+00	6.640E+00	6.732E+00	6.264E+00	5.309E+00	4.154E+00	2.907E+00	2.527E+00
0.1	6.300E+00	6.665E+00	6.702E+00	6.268E+00	5.359E+00	4.129E+00	2.897E+00	2.506E+00
0.15	6.364E+00	6.678E+00	6.790E+00	6.282E+00	5.347E+00	4.146E+00	2.927E+00	2.555E+00
0.2	6.426E+00	6.780E+00	6.853E+00	6.425E+00	5.456E+00	4.243E+00	2.939E+00	2.577E+00
0.3	6.565E+00	6.933E+00	7.020E+00	6.460E+00	5.482E+00	4.336E+00	2.982E+00	2.668E+00
0.4	6.762E+00	7.111E+00	7.163E+00	6.574E+00	5.620E+00	4.405E+00	3.070E+00	2.720E+00
0.5	6.978E+00	7.358E+00	7.418E+00	6.754E+00	5.822E+00	4.454E+00	3.164E+00	2.799E+00
0.6	7.308E+00	7.601E+00	7.675E+00	7.040E+00	5.945E+00	4.674E+00	3.245E+00	2.909E+00
0.7	9.185E+00	9.704E+00	9.909E+00	9.152E+00	7.396E+00	5.371E+00	3.507E+00	3.681E+00
0.8	1.893E+01	1.940E+01	1.956E+01	1.726E+01	1.315E+01	8.522E+00	4.684E+00	6.867E+00
1	8.224E+01	8.016E+01	7.231E+01	5.913E+01	4.202E+01	2.511E+01	1.197E+01	2.529E+01
1.25	2.052E+02	1.994E+02	1.760E+02	1.440E+02	1.025E+02	6.128E+01	2.900E+01	6.241E+01
1.5	3.167E+02	3.074E+02	2.775E+02	2.320E+02	1.700E+02	1.047E+02	5.253E+01	9.914E+01
1.75	3.836E+02	3.770E+02	3.512E+02	3.069E+02	2.347E+02	1.524E+02	7.858E+01	1.270E+02
2	4.064E+02	4.034E+02	3.875E+02	3.534E+02	2.881E+02	1.976E+02	1.082E+02	1.440E+02
2.5	3.878E+02	3.941E+02	3.972E+02	3.889E+02	3.492E+02	2.662E+02	1.646E+02	1.568E+02
3	3.595E+02	3.685E+02	3.785E+02	3.963E+02	3.687E+02	3.119E+02	2.117E+02	1.588E+02
3.5	3.377E+02	3.472E+02	3.649E+02	3.845E+02	3.735E+02	3.400E+02	2.479E+02	1.573E+02
4	3.222E+02	3.362E+02	3.533E+02	3.805E+02	3.683E+02	3.552E+02	2.784E+02	1.582E+02
5	3.103E+02	3.216E+02	3.423E+02	3.743E+02	3.493E+02	3.651E+02	3.229E+02	1.604E+02
6	3.046E+02	3.173E+02	3.384E+02	3.803E+02	3.357E+02	3.595E+02	3.476E+02	1.625E+02
7	3.001E+02	3.123E+02	3.344E+02	3.836E+02	3.252E+02	3.458E+02	3.592E+02	1.674E+02
8	2.987E+02	3.058E+02	3.324E+02	3.910E+02	3.158E+02	3.384E+02	3.606E+02	1.702E+02
10	2.962E+02	3.019E+02	3.229E+02	3.882E+02	3.330E+02	3.235E+02	3.445E+02	1.798E+02
15	2.973E+02	3.007E+02	3.058E+02	3.402E+02	3.940E+02	3.095E+02	3.169E+02	1.943E+02
20	2.980E+02	2.992E+02	3.010E+02	3.159E+02	3.865E+02	3.583E+02	3.047E+02	2.066E+02
30	2.989E+02	2.991E+02	2.996E+02	3.075E+02	3.530E+02	3.472E+02	3.032E+02	2.309E+02
40	2.987E+02	3.026E+02	3.026E+02	3.030E+02	3.396E+02	3.302E+02	3.010E+02	2.598E+02
50	3.017E+02	2.995E+02	3.032E+02	3.022E+02	3.306E+02	3.226E+02	3.026E+02	2.828E+02

**Table A9.** Absorbed dose per photon fluence (CPE) for the complete lens, *D*_lens_/*Φ*, for mono-energetic particles incident at different angles *α* and the ROT geometry. Each value is the maximum value of the two lenses; except for 0° and ROT, the mean of the two lenses is given.

Photon energy (MeV)	Absorbed dose per fluence to the radiation sensitive part of the lens, *D*_lens,s_/*Φ*, (pGy cm²) for a radiation incidence at *α*							ROT geometry
	0°	15°	30°	45°	60°	75°	90°	
0.005	8.428E−06	1.564E−05	3.251E−05	3.233E−05	1.203E−05	1.594E−06	8.802E−08	6.675E−06
0.006	2.010E−03	2.336E−03	2.972E−03	2.760E−03	1.461E−03	3.826E−04	4.322E−05	8.424E−04
0.007	3.470E−02	3.552E−02	3.654E−02	3.189E−02	2.034E−02	7.951E−03	1.438E−03	1.154E−02
0.008	1.732E−01	1.717E−01	1.638E−01	1.406E−01	9.777E−02	4.718E−02	1.350E−02	5.447E−02
0.009	4.489E−01	4.401E−01	4.118E−01	3.563E−01	2.667E−01	1.510E−01	5.484E−02	1.436E−01
0.01	7.958E−01	7.776E−01	7.302E−01	6.443E−01	5.084E−01	3.217E−01	1.394E−01	2.635E−01
0.011	1.123E+00	1.104E+00	1.043E+00	9.342E−01	7.721E−01	5.361E−01	2.699E−01	3.871E−01
0.013	1.538E+00	1.518E+00	1.459E+00	1.352E+00	1.191E+00	9.338E−01	5.786E−01	5.750E−01
0.015	1.647E+00	1.629E+00	1.581E+00	1.503E+00	1.377E+00	1.163E+00	8.295E−01	6.596E−01
0.017	1.580E+00	1.570E+00	1.536E+00	1.482E+00	1.390E+00	1.225E+00	9.581E−01	6.727E−01
0.02	1.381E+00	1.376E+00	1.356E+00	1.321E+00	1.269E+00	1.165E+00	9.824E−01	6.284E−01
0.024	1.112E+00	1.116E+00	1.110E+00	1.092E+00	1.063E+00	9.995E−01	8.873E−01	5.464E−01
0.03	8.315E−01	8.355E−01	8.388E−01	8.316E−01	8.112E−01	7.782E−01	7.173E−01	4.408E−01
0.04	5.868E−01	5.950E−01	5.964E−01	5.961E−01	5.900E−01	5.706E−01	5.353E−01	3.397E−01
0.05	4.883E−01	4.895E−01	4.983E−01	4.988E−01	4.954E−01	4.791E−01	4.533E−01	2.975E−01
0.06	4.550E−01	4.563E−01	4.642E−01	4.665E−01	4.619E−01	4.463E−01	4.272E−01	2.857E−01
0.07	4.559E−01	4.623E−01	4.658E−01	4.673E−01	4.637E−01	4.524E−01	4.341E−01	2.953E−01
0.08	4.806E−01	4.869E−01	4.911E−01	4.904E−01	4.877E−01	4.778E−01	4.601E−01	3.155E−01
0.1	5.593E−01	5.622E−01	5.722E−01	5.710E−01	5.646E−01	5.586E−01	5.409E−01	3.766E−01
0.12	6.637E−01	6.673E−01	6.730E−01	6.741E−01	6.716E−01	6.662E−01	6.437E−01	4.522E−01
0.15	8.351E−01	8.385E−01	8.461E−01	8.497E−01	8.446E−01	8.392E−01	8.192E−01	5.811E−01
0.2	1.133E+00	1.140E+00	1.153E+00	1.169E+00	1.147E+00	1.146E+00	1.126E+00	8.171E−01
0.24	1.377E+00	1.389E+00	1.395E+00	1.409E+00	1.391E+00	1.382E+00	1.375E+00	9.996E−01
0.3	1.741E+00	1.742E+00	1.758E+00	1.789E+00	1.751E+00	1.746E+00	1.727E+00	1.292E+00
0.4	2.303E+00	2.336E+00	2.342E+00	2.372E+00	2.338E+00	2.318E+00	2.291E+00	1.753E+00
0.5	2.821E+00	2.848E+00	2.877E+00	2.932E+00	2.902E+00	2.836E+00	2.850E+00	2.211E+00
0.511	2.883E+00	2.900E+00	2.938E+00	2.992E+00	2.957E+00	2.902E+00	2.911E+00	2.253E+00
0.6	3.328E+00	3.350E+00	3.394E+00	3.446E+00	3.419E+00	3.350E+00	3.364E+00	2.642E+00
0.662	3.634E+00	3.647E+00	3.689E+00	3.770E+00	3.725E+00	3.648E+00	3.652E+00	2.909E+00
0.8	4.245E+00	4.278E+00	4.302E+00	4.390E+00	4.381E+00	4.281E+00	4.288E+00	3.460E+00
1	5.084E+00	5.099E+00	5.150E+00	5.253E+00	5.228E+00	5.126E+00	5.124E+00	4.206E+00
1.117	5.537E+00	5.573E+00	5.631E+00	5.686E+00	5.676E+00	5.549E+00	5.565E+00	4.626E+00
1.2	5.831E+00	5.913E+00	5.944E+00	5.992E+00	5.965E+00	5.893E+00	5.876E+00	4.917E+00
1.3	6.198E+00	6.208E+00	6.275E+00	6.371E+00	6.355E+00	6.224E+00	6.253E+00	5.239E+00
1.33	6.289E+00	6.308E+00	6.365E+00	6.462E+00	6.440E+00	6.338E+00	6.339E+00	5.358E+00
1.5	6.875E+00	6.901E+00	6.919E+00	7.043E+00	7.046E+00	6.862E+00	6.897E+00	5.865E+00
1.7	7.487E+00	7.526E+00	7.577E+00	7.668E+00	7.647E+00	7.488E+00	7.544E+00	6.477E+00
2	8.386E+00	8.387E+00	8.443E+00	8.556E+00	8.527E+00	8.407E+00	8.373E+00	7.252E+00
2.4	9.443E+00	9.493E+00	9.504E+00	9.632E+00	9.644E+00	9.478E+00	9.526E+00	8.330E+00
3	1.096E+01	1.102E+01	1.104E+01	1.112E+01	1.109E+01	1.098E+01	1.091E+01	9.772E+00
4	1.325E+01	1.324E+01	1.333E+01	1.334E+01	1.331E+01	1.315E+01	1.316E+01	1.186E+01
5	1.533E+01	1.533E+01	1.536E+01	1.544E+01	1.549E+01	1.534E+01	1.533E+01	1.390E+01
6	1.738E+01	1.744E+01	1.741E+01	1.748E+01	1.750E+01	1.737E+01	1.740E+01	1.585E+01
6.129	1.760E+01	1.769E+01	1.765E+01	1.780E+01	1.781E+01	1.759E+01	1.759E+01	1.609E+01
8	2.147E+01	2.139E+01	2.152E+01	2.148E+01	2.148E+01	2.139E+01	2.132E+01	1.944E+01
10	2.536E+01	2.541E+01	2.554E+01	2.543E+01	2.544E+01	2.535E+01	2.536E+01	2.347E+01
15	3.570E+01	3.580E+01	3.560E+01	3.560E+01	3.569E+01	3.555E+01	3.564E+01	3.282E+01
20	4.627E+01	4.656E+01	4.655E+01	4.663E+01	4.645E+01	4.633E+01	4.617E+01	4.298E+01
30	6.922E+01	6.910E+01	6.931E+01	6.911E+01	6.939E+01	6.912E+01	6.859E+01	6.390E+01
40	9.300E+01	9.350E+01	9.326E+01	9.275E+01	9.315E+01	9.323E+01	9.229E+01	8.634E+01
50	1.182E+02	1.191E+02	1.187E+02	1.184E+02	1.178E+02	1.181E+02	1.178E+02	1.094E+02

**Table A10.** Absorbed dose per photon fluence (non-CPE) for the complete lens, *D*_lens_/*Φ*, for mono-energetic particles incident at different angles *α* and the ROT geometry. Each value is the maximum value of the two lenses; except for 0° and ROT, the mean of the two lenses is given.

Photon energy (MeV)	Absorbed dose per fluence to the radiation sensitive part of the lens, *D*_lens,s_/*Φ*, (pGy cm²) for a radiation incidence at *α*							ROT geometry
	0°	15°	30°	45°	60°	75°	90°	
0.005	8.605E−06	1.625E−05	3.469E−05	3.293E−05	1.346E−05	1.660E−06	6.897E−08	8.854E−06
0.006	1.999E−03	2.444E−03	2.973E−03	2.854E−03	1.481E−03	4.077E−04	4.262E−05	8.746E−04
0.007	3.738E−02	3.556E−02	3.668E−02	3.378E−02	2.018E−02	7.751E−03	1.754E−03	1.239E−02
0.008	1.846E−01	1.717E−01	1.629E−01	1.496E−01	9.809E−02	4.696E−02	1.467E−02	5.806E−02
0.009	4.749E−01	4.412E−01	4.126E−01	3.768E−01	2.662E−01	1.499E−01	5.893E−02	1.502E−01
0.01	8.330E−01	7.776E−01	7.301E−01	6.724E−01	5.071E−01	3.204E−01	1.523E−01	2.738E−01
0.011	1.154E+00	1.102E+00	1.040E+00	9.657E−01	7.715E−01	5.352E−01	2.890E−01	3.980E−01
0.013	1.536E+00	1.519E+00	1.459E+00	1.355E+00	1.194E+00	9.318E−01	5.981E−01	5.796E−01
0.015	1.625E+00	1.628E+00	1.583E+00	1.486E+00	1.375E+00	1.161E+00	8.310E−01	6.575E−01
0.017	1.550E+00	1.572E+00	1.536E+00	1.460E+00	1.390E+00	1.229E+00	9.526E−01	6.659E−01
0.02	1.345E+00	1.373E+00	1.359E+00	1.298E+00	1.271E+00	1.164E+00	9.649E−01	6.193E−01
0.024	1.089E+00	1.109E+00	1.112E+00	1.068E+00	1.059E+00	9.975E−01	8.693E−01	5.354E−01
0.03	8.116E−01	8.350E−01	8.339E−01	8.127E−01	8.132E−01	7.804E−01	6.995E−01	4.342E−01
0.04	5.805E−01	5.926E−01	6.011E−01	5.895E−01	5.924E−01	5.692E−01	5.289E−01	3.365E−01
0.05	4.832E−01	4.939E−01	5.035E−01	4.964E−01	4.946E−01	4.765E−01	4.468E−01	2.946E−01
0.06	4.497E−01	4.594E−01	4.666E−01	4.640E−01	4.612E−01	4.500E−01	4.277E−01	2.849E−01
0.07	4.554E−01	4.627E−01	4.666E−01	4.632E−01	4.614E−01	4.534E−01	4.334E−01	2.935E−01
0.08	4.818E−01	4.835E−01	4.888E−01	4.850E−01	4.900E−01	4.794E−01	4.584E−01	3.146E−01
0.1	5.592E−01	5.620E−01	5.692E−01	5.706E−01	5.703E−01	5.573E−01	5.406E−01	3.756E−01
0.12	6.629E−01	6.656E−01	6.723E−01	6.728E−01	6.707E−01	6.627E−01	6.434E−01	4.519E−01
0.15	8.378E−01	8.402E−01	8.459E−01	8.451E−01	8.450E−01	8.355E−01	8.108E−01	5.796E−01
0.2	1.134E+00	1.147E+00	1.163E+00	1.170E+00	1.147E+00	1.139E+00	1.128E+00	8.096E−01
0.24	1.379E+00	1.382E+00	1.407E+00	1.417E+00	1.391E+00	1.376E+00	1.373E+00	1.003E+00
0.3	1.737E+00	1.748E+00	1.768E+00	1.805E+00	1.751E+00	1.746E+00	1.731E+00	1.284E+00
0.4	2.295E+00	2.319E+00	2.338E+00	2.376E+00	2.349E+00	2.313E+00	2.324E+00	1.754E+00
0.5	2.828E+00	2.836E+00	2.889E+00	2.926E+00	2.904E+00	2.841E+00	2.834E+00	2.216E+00
0.511	2.877E+00	2.880E+00	2.973E+00	3.012E+00	2.985E+00	2.887E+00	2.892E+00	2.255E+00
0.6	3.343E+00	3.362E+00	3.402E+00	3.461E+00	3.413E+00	3.356E+00	3.348E+00	2.638E+00
0.662	3.628E+00	3.647E+00	3.663E+00	3.766E+00	3.704E+00	3.655E+00	3.644E+00	2.896E+00
0.8	4.264E+00	4.282E+00	4.327E+00	4.393E+00	4.369E+00	4.275E+00	4.265E+00	3.461E+00
1	5.057E+00	5.085E+00	5.142E+00	5.275E+00	5.207E+00	5.076E+00	5.121E+00	4.196E+00
1.117	5.500E+00	5.546E+00	5.563E+00	5.647E+00	5.639E+00	5.561E+00	5.567E+00	4.631E+00
1.2	5.835E+00	5.845E+00	5.864E+00	5.983E+00	5.950E+00	5.824E+00	5.925E+00	4.867E+00
1.3	6.074E+00	6.140E+00	6.165E+00	6.352E+00	6.296E+00	6.148E+00	6.199E+00	5.182E+00
1.33	6.165E+00	6.258E+00	6.257E+00	6.400E+00	6.451E+00	6.289E+00	6.290E+00	5.253E+00
1.5	6.590E+00	6.630E+00	6.710E+00	6.879E+00	6.905E+00	6.735E+00	6.834E+00	5.757E+00
1.7	6.921E+00	6.927E+00	7.084E+00	7.255E+00	7.396E+00	7.233E+00	7.374E+00	6.200E+00
2	7.045E+00	7.162E+00	7.286E+00	7.657E+00	7.920E+00	7.877E+00	8.050E+00	6.747E+00
2.4	6.844E+00	6.933E+00	7.243E+00	7.840E+00	8.467E+00	8.644E+00	8.737E+00	7.315E+00
3	6.349E+00	6.492E+00	6.918E+00	7.868E+00	8.995E+00	9.607E+00	9.324E+00	7.860E+00
4	5.624E+00	5.846E+00	6.427E+00	7.674E+00	9.641E+00	1.094E+01	1.097E+01	8.626E+00
5	5.129E+00	5.350E+00	6.080E+00	7.676E+00	1.014E+01	1.221E+01	1.261E+01	9.355E+00
6	4.822E+00	5.045E+00	5.869E+00	7.597E+00	1.071E+01	1.342E+01	1.404E+01	9.994E+00
6.129	4.795E+00	5.061E+00	5.762E+00	7.617E+00	1.076E+01	1.336E+01	1.428E+01	1.011E+01
8	4.424E+00	4.667E+00	5.516E+00	7.467E+00	1.156E+01	1.557E+01	1.706E+01	1.141E+01
10	4.168E+00	4.376E+00	5.194E+00	7.164E+00	1.221E+01	1.756E+01	1.947E+01	1.270E+01
15	3.973E+00	4.161E+00	4.776E+00	6.578E+00	1.249E+01	2.081E+01	2.573E+01	1.582E+01
20	3.943E+00	4.083E+00	4.604E+00	6.146E+00	1.244E+01	2.286E+01	3.088E+01	1.892E+01
30	4.006E+00	4.118E+00	4.585E+00	5.837E+00	1.192E+01	2.459E+01	3.794E+01	2.446E+01
40	4.091E+00	4.184E+00	4.676E+00	5.692E+00	1.171E+01	2.546E+01	4.221E+01	2.944E+01
50	4.159E+00	4.319E+00	4.711E+00	5.705E+00	1.164E+01	2.636E+01	4.529E+01	3.358E+01

**Table A11.** Absorbed dose per photon air kerma (CPE) for the complete lens, *D*_lens_/*K*_a_, for mono-energetic particles incident at different angles *α* and the ROT geometry. Each value is the maximum value of the two lenses; except for 0° and ROT, the mean of the two lenses is given.

Photon energy (MeV)	Absorbed dose per air kerma to the radiation sensitive part of the lens, *D*_lens,s_/*K*_a_, (Gy / Gy) for a radiation incidence at *α*							ROT geometry
	0°	15°	30°	45°	60°	75°	90°	
0.005	2.677E−07	4.968E−07	1.032E−06	1.027E−06	3.821E−07	5.062E−08	2.795E−09	2.120E−07
0.006	9.211E−05	1.070E−04	1.362E−04	1.265E−04	6.695E−05	1.753E−05	1.981E−06	3.861E−05
0.007	2.180E−03	2.232E−03	2.296E−03	2.004E−03	1.278E−03	4.996E−04	9.032E−05	7.250E−04
0.008	1.431E−02	1.418E−02	1.352E−02	1.161E−02	8.073E−03	3.896E−03	1.114E−03	4.498E−03
0.009	4.741E−02	4.649E−02	4.350E−02	3.763E−02	2.817E−02	1.595E−02	5.792E−03	1.517E−02
0.01	1.048E−01	1.024E−01	9.612E−02	8.481E−02	6.692E−02	4.235E−02	1.835E−02	3.468E−02
0.011	1.811E−01	1.780E−01	1.681E−01	1.506E−01	1.245E−01	8.643E−02	4.352E−02	6.240E−02
0.013	3.537E−01	3.492E−01	3.355E−01	3.111E−01	2.740E−01	2.148E−01	1.331E−01	1.323E−01
0.015	5.137E−01	5.082E−01	4.932E−01	4.688E−01	4.294E−01	3.628E−01	2.587E−01	2.057E−01
0.017	6.452E−01	6.410E−01	6.272E−01	6.052E−01	5.675E−01	5.002E−01	3.911E−01	2.746E−01
0.02	7.996E−01	7.965E−01	7.855E−01	7.652E−01	7.346E−01	6.747E−01	5.688E−01	3.639E−01
0.024	9.431E−01	9.464E−01	9.420E−01	9.262E−01	9.021E−01	8.479E−01	7.527E−01	4.635E−01
0.03	1.126E+00	1.131E+00	1.136E+00	1.126E+00	1.098E+00	1.054E+00	9.710E−01	5.967E−01
0.04	1.340E+00	1.359E+00	1.362E+00	1.361E+00	1.347E+00	1.303E+00	1.222E+00	7.758E−01
0.05	1.487E+00	1.491E+00	1.518E+00	1.519E+00	1.509E+00	1.459E+00	1.381E+00	9.063E−01
0.06	1.557E+00	1.561E+00	1.588E+00	1.596E+00	1.580E+00	1.527E+00	1.462E+00	9.773E−01
0.07	1.571E+00	1.593E+00	1.605E+00	1.610E+00	1.598E+00	1.559E+00	1.496E+00	1.018E+00
0.08	1.558E+00	1.578E+00	1.592E+00	1.590E+00	1.581E+00	1.549E+00	1.491E+00	1.023E+00
0.1	1.502E+00	1.509E+00	1.536E+00	1.533E+00	1.516E+00	1.500E+00	1.452E+00	1.011E+00
0.12	1.438E+00	1.446E+00	1.458E+00	1.461E+00	1.455E+00	1.444E+00	1.395E+00	9.798E−01
0.15	1.392E+00	1.398E+00	1.411E+00	1.417E+00	1.408E+00	1.399E+00	1.366E+00	9.689E−01
0.2	1.323E+00	1.331E+00	1.347E+00	1.366E+00	1.340E+00	1.339E+00	1.315E+00	9.543E−01
0.24	1.297E+00	1.309E+00	1.315E+00	1.327E+00	1.311E+00	1.303E+00	1.295E+00	9.419E−01
0.3	1.262E+00	1.263E+00	1.274E+00	1.296E+00	1.269E+00	1.265E+00	1.251E+00	9.360E−01
0.4	1.219E+00	1.236E+00	1.239E+00	1.255E+00	1.237E+00	1.226E+00	1.212E+00	9.275E−01
0.5	1.187E+00	1.199E+00	1.211E+00	1.234E+00	1.221E+00	1.194E+00	1.199E+00	9.304E−01
0.511	1.188E+00	1.195E+00	1.210E+00	1.233E+00	1.219E+00	1.196E+00	1.200E+00	9.281E−01
0.6	1.172E+00	1.180E+00	1.196E+00	1.214E+00	1.204E+00	1.180E+00	1.185E+00	9.305E−01
0.662	1.170E+00	1.174E+00	1.188E+00	1.214E+00	1.199E+00	1.175E+00	1.176E+00	9.367E−01
0.8	1.149E+00	1.158E+00	1.165E+00	1.188E+00	1.186E+00	1.159E+00	1.161E+00	9.367E−01
1	1.138E+00	1.141E+00	1.153E+00	1.176E+00	1.170E+00	1.147E+00	1.147E+00	9.413E−01
1.117	1.137E+00	1.145E+00	1.157E+00	1.168E+00	1.166E+00	1.140E+00	1.143E+00	9.500E−01
1.2	1.133E+00	1.149E+00	1.155E+00	1.164E+00	1.159E+00	1.145E+00	1.142E+00	9.553E−01
1.3	1.132E+00	1.134E+00	1.146E+00	1.163E+00	1.160E+00	1.136E+00	1.142E+00	9.567E−01
1.33	1.128E+00	1.132E+00	1.142E+00	1.159E+00	1.155E+00	1.137E+00	1.137E+00	9.612E−01
1.5	1.123E+00	1.128E+00	1.130E+00	1.151E+00	1.151E+00	1.121E+00	1.127E+00	9.583E−01
1.7	1.119E+00	1.125E+00	1.132E+00	1.146E+00	1.143E+00	1.119E+00	1.127E+00	9.679E−01
2	1.116E+00	1.116E+00	1.124E+00	1.139E+00	1.135E+00	1.119E+00	1.114E+00	9.652E−01
2.4	1.111E+00	1.117E+00	1.118E+00	1.133E+00	1.134E+00	1.115E+00	1.121E+00	9.799E−01
3	1.109E+00	1.114E+00	1.116E+00	1.125E+00	1.121E+00	1.110E+00	1.104E+00	9.884E−01
4	1.106E+00	1.105E+00	1.113E+00	1.113E+00	1.111E+00	1.098E+00	1.098E+00	9.897E−01
5	1.100E+00	1.100E+00	1.102E+00	1.108E+00	1.111E+00	1.101E+00	1.100E+00	9.971E−01
6	1.098E+00	1.102E+00	1.100E+00	1.104E+00	1.106E+00	1.097E+00	1.099E+00	1.001E+00
6.129	1.095E+00	1.100E+00	1.098E+00	1.107E+00	1.107E+00	1.094E+00	1.094E+00	1.001E+00
8	1.098E+00	1.094E+00	1.101E+00	1.099E+00	1.099E+00	1.094E+00	1.091E+00	9.943E−01
10	1.092E+00	1.094E+00	1.099E+00	1.095E+00	1.095E+00	1.091E+00	1.092E+00	1.010E+00
15	1.098E+00	1.101E+00	1.095E+00	1.095E+00	1.098E+00	1.093E+00	1.096E+00	1.010E+00
20	1.101E+00	1.108E+00	1.108E+00	1.110E+00	1.106E+00	1.103E+00	1.099E+00	1.023E+00
30	1.128E+00	1.126E+00	1.129E+00	1.126E+00	1.130E+00	1.126E+00	1.118E+00	1.041E+00
40	1.150E+00	1.156E+00	1.153E+00	1.147E+00	1.152E+00	1.153E+00	1.141E+00	1.068E+00
50	1.179E+00	1.187E+00	1.184E+00	1.181E+00	1.175E+00	1.177E+00	1.175E+00	1.091E+00

**Table A12.** Absorbed dose per photon air kerma (non-CPE) for the complete lens, *D*_lens_/*K*_a_, for mono-energetic particles incident at different angles *α* and the ROT geometry. Each value is the maximum value of the two lenses; except for 0° and ROT, the mean of the two lenses is given.

Photon energy (MeV)	Absorbed dose per air kerma to the radiation sensitive part of the lens, *D*_lens,s_/*K*_a_, (Gy/Gy) for a radiation incidence at *α*							ROT geometry
	0°	15°	30°	45°	60°	75°	90°	
0.005	2.733E−07	5.161E−07	1.102E−06	1.046E−06	4.273E−07	5.272E−08	2.190E−09	2.812E−07
0.006	9.163E−05	1.120E−04	1.362E−04	1.308E−04	6.788E−05	1.869E−05	1.953E−06	4.008E−05
0.007	2.348E−03	2.234E−03	2.305E−03	2.123E−03	1.268E−03	4.870E−04	1.102E−04	7.786E−04
0.008	1.525E−02	1.418E−02	1.345E−02	1.236E−02	8.100E−03	3.878E−03	1.212E−03	4.795E−03
0.009	5.016E−02	4.660E−02	4.358E−02	3.979E−02	2.811E−02	1.583E−02	6.224E−03	1.586E−02
0.01	1.097E−01	1.024E−01	9.610E−02	8.851E−02	6.675E−02	4.217E−02	2.005E−02	3.604E−02
0.011	1.861E−01	1.776E−01	1.676E−01	1.557E−01	1.244E−01	8.629E−02	4.659E−02	6.417E−02
0.013	3.534E−01	3.495E−01	3.356E−01	3.116E−01	2.746E−01	2.143E−01	1.376E−01	1.333E−01
0.015	5.069E−01	5.076E−01	4.938E−01	4.635E−01	4.288E−01	3.621E−01	2.592E−01	2.05101
0.017	6.329E−01	6.419E−01	6.270E−01	5.959E−01	5.675E−01	5.016E−01	3.889E−01	2.719E−01
0.02	7.788E−01	7.949E−01	7.871E−01	7.515E−01	7.362E−01	6.742E−01	5.587E−01	3.586E−01
0.024	9.236E−01	9.408E−01	9.429E−01	9.063E−01	8.981E−01	8.462E−01	7.374E−01	4.542E−01
0.03	1.099E+00	1.130E+00	1.129E+00	1.100E+00	1.101E+00	1.056E+00	9.469E−01	5.878E−01
0.04	1.326E+00	1.353E+00	1.373E+00	1.346E+00	1.353E+00	1.300E+00	1.208E+00	7.684E−01
0.05	1.472E+00	1.504E+00	1.534E+00	1.512E+00	1.507E+00	1.451E+00	1.361E+00	8.975E−01
0.06	1.538E+00	1.572E+00	1.596E+00	1.588E+00	1.578E+00	1.540E+00	1.463E+00	9.747E−01
0.07	1.569E+00	1.595E+00	1.608E+00	1.596E+00	1.590E+00	1.563E+00	1.494E+00	1.012E+00
0.08	1.562E+00	1.567E+00	1.584E+00	1.572E+00	1.588E+00	1.554E+00	1.486E+00	1.020E+00
0.1	1.501E+00	1.509E+00	1.528E+00	1.532E+00	1.531E+00	1.496E+00	1.451E+00	1.008E+00
0.12	1.437E+00	1.442E+00	1.457E+00	1.458E+00	1.453E+00	1.436E+00	1.394E+00	9.792E−01
0.15	1.397E+00	1.401E+00	1.410E+00	1.409E+00	1.409E+00	1.393E+00	1.352E+00	9.664E−01
0.2	1.325E+00	1.340E+00	1.358E+00	1.366E+00	1.339E+00	1.330E+00	1.318E+00	9.455E−01
0.24	1.299E+00	1.302E+00	1.325E+00	1.336E+00	1.311E+00	1.296E+00	1.294E+00	9.450E−01
0.3	1.259E+00	1.266E+00	1.281E+00	1.308E+00	1.269E+00	1.266E+00	1.254E+00	9.302E−01
0.4	1.214E+00	1.227E+00	1.237E+00	1.257E+00	1.243E+00	1.224E+00	1.230E+00	9.283E−01
0.5	1.190E+00	1.193E+00	1.216E+00	1.232E+00	1.222E+00	1.196E+00	1.193E+00	9.325E−01
0.511	1.185E+00	1.187E+00	1.225E+00	1.241E+00	1.230E+00	1.189E+00	1.192E+00	9.293E−01
0.6	1.178E+00	1.184E+00	1.198E+00	1.219E+00	1.202E+00	1.182E+00	1.179E+00	9.291E−01
0.662	1.168E+00	1.174E+00	1.179E+00	1.213E+00	1.192E+00	1.177E+00	1.173E+00	9.325E−01
0.8	1.154E+00	1.159E+00	1.171E+00	1.189E+00	1.183E+00	1.157E+00	1.155E+00	9.370E−01
1	1.132E+00	1.138E+00	1.151E+00	1.181E+00	1.165E+00	1.136E+00	1.146E+00	9.390E−01
1.117	1.130E+00	1.139E+00	1.143E+00	1.160E+00	1.158E+00	1.142E+00	1.143E+00	9.512E−01
1.2	1.134E+00	1.136E+00	1.139E+00	1.162E+00	1.156E+00	1.132E+00	1.151E+00	9.456E−01
1.3	1.109E+00	1.121E+00	1.126E+00	1.160E+00	1.150E+00	1.123E+00	1.132E+00	9.461E−01
1.33	1.106E+00	1.123E+00	1.122E+00	1.148E+00	1.157E+00	1.128E+00	1.128E+00	9.424E−01
1.5	1.077E+00	1.083E+00	1.096E+00	1.124E+00	1.128E+00	1.101E+00	1.117E+00	9.406E−01
1.7	1.034E+00	1.035E+00	1.059E+00	1.084E+00	1.105E+00	1.081E+00	1.102E+00	9.265E−01
2	9.375E−01	9.531E−01	9.697E−01	1.019E+00	1.054E+00	1.048E+00	1.071E+00	8.980E−01
2.4	8.051E−01	8.155E−01	8.520E−01	9.222E−01	9.960E−01	1.017E+00	1.028E+00	8.605E−01
3	6.422E−01	6.566E−01	6.997E−01	7.958E−01	9.097E−01	9.716E−01	9.431E−01	7.950E−01
4	4.694E−01	4.880E−01	5.365E−01	6.406E−01	8.047E−01	9.128E−01	9.157E−01	7.200E−01
5	3.679E−01	3.838E−01	4.362E−01	5.506E−01	7.275E−01	8.759E−01	9.046E−01	6.711E−01
6	3.046E−01	3.187E−01	3.707E−01	4.799E−01	6.766E−01	8.478E−01	8.868E−01	6.313E−01
6.129	2.982E−01	3.147E−01	3.584E−01	4.737E−01	6.691E−01	8.306E−01	8.879E−01	6.289E−01
8	2.263E−01	2.387E−01	2.822E−01	3.820E−01	5.914E−01	7.965E−01	8.727E−01	5.837E−01
10	1.794E−01	1.884E−01	2.236E−01	3.084E−01	5.258E−01	7.559E−01	8.382E−01	5.466E−01
15	1.222E−01	1.280E−01	1.469E−01	2.023E−01	3.842E−01	6.401E−01	7.915E−01	4.867E−01
20	9.386E−02	9.719E−02	1.096E−01	1.463E−01	2.961E−01	5.441E−01	7.350E−01	4.504E−01
30	6.526E−02	6.708E−02	7.469E−02	9.510E−02	1.942E−01	4.006E−01	6.181E−01	3.986E−01
40	5.058E−02	5.174E−02	5.782E−02	7.039E−02	1.448E−01	3.149E−01	5.220E−01	3.640E−01
50	4.147E−02	4.307E−02	4.697E−02	5.688E−02	1.161E−01	2.628E−01	4.515E−01	3.348E−01

All data sets (including the statistical uncertainties) can be downloaded from this article's website. The online data set contains separate files for the left and right lens and for the maximum values of the two lenses (as in Tables A1–A12) for both the sensitive cells and the complete lens and, in addition, for the the maximum of the left and the right lens of both the sensitive cells and the complete lens (i.e. the maximum of these four values).

Interpolation between values at given energies should be performed using a log–log interpolation.
